# Maximizing completeness in single-crystal high-pressure diffraction experiments: phase transitions in 2°AP

**DOI:** 10.1107/S2052252521009532

**Published:** 2021-10-15

**Authors:** D. Tchoń, A. Makal

**Affiliations:** a Biological and Chemical Research Centre, Faculty of Chemistry, University of Warsaw, Żwirki i Wigury 101, 02-089 Warszawa, Poland

**Keywords:** diamond anvil cell, molecular crystals, phase transitions, high-pressure XRD, polymorphism, properties of solids, X-ray data completeness

## Abstract

Sufficiently high completeness of diffraction data is necessary to correctly determine the space group, observe solid-state structural transformations or investigate charge density distribution under pressure. Regrettably, experiments performed at high pressure in a diamond anvil cell (DAC) yield inherently incomplete datasets. The present work systematizes the combined influence of radiation wavelength, DAC opening angle and sample orientation in a DAC on the completeness of diffraction data collected in a single-crystal high-pressure (HP) experiment with the help of dedicated software. In particular, the impact of the sample orientation on the achievable data completeness is quantified and proved to be substantial. Graphical guides for estimating the most beneficial sample orientation depending on the sample Laue class and assuming a few commonly used experimental setups are proposed. The usefulness of these guides has been tested in the case of luminescent 1,3-diacetylpyrene, suspected to undergo transitions from the α phase (*Pnma*) to the γ phase (*Pn*2_1_ *a*) and δ phase (*P*112_1_/*a*) under pressure. Effective sample orientation has ensured over 90% coverage even for the monoclinic system and enabled unrestrained structure refinements and access to complete systematic extinction patterns.

## Abstract 2

The influence of a diamond anvil cell (DAC) aperture, incident radiation wavelength and sample orientation in a DAC on the completeness of diffraction data collected at high pressure has been systematically analyzed. The considerable impact of sample orientation on achievable data completeness has been confirmed and quantified for all Laue classes in the form of graphical guides. These guides have been applied to predict sample orientation for structural analysis of a sample undergoing an orthorhombic → monoclinic phase transition under pressure which ensured over 90% completeness.

## 1. Introduction   

Insufficient reciprocal space coverage in diffraction experiments may impede space group determination (Merli *et al.*, 2002 ▸; Friese & Grzechnik, 2014 ▸; Sheldrick, 2015*a* ▸), render solution of a crystal structure (Yogavel *et al.*, 2007 ▸) or determination of absolute configuration (Friese & Grzechnik, 2014 ▸) impossible, and conceal or misrepresent fine details such as disorder or unusual charge density distribution (Takata & Sakata, 1996 ▸). Data completeness of 40% or below generally poses difficulties for most structure-solution methods and results in numerous artifacts on Fourier maps. Standardized quality checks demand that diffraction patterns be complete up to a certain resolution, traditionally 0.6 Å^−1^ (Spek, 2020 ▸).

Although in the majority of X-ray diffraction experiments modern area detectors and multi-axis goniometers allow all reflections to be recorded, the signal will not be observed if the beam does not have access to the sample altogether. This is particularly the case when a diamond anvil cell (DAC) is used in HP structural analysis (Merrill & Bassett, 1974 ▸; Dziubek & Katrusiak, 2002 ▸; Dawson *et al.*, 2004 ▸).

Crystallographic studies at high pressure have, over the years, developed into a powerful technique which can be applied to tackle numerous scientific problems (Katrusiak, 2019 ▸). Recent publications indicate a variety of applications in the analysis of phase diagrams, polymorphism and relative stability of pharmaceutical compounds (Guerain, 2020 ▸); investigation of structure-property relationships in metal-organic frameworks (McKellar & Moggach, 2015 ▸); reproducing certain geochemical processes (Xu *et al.*, 2020 ▸; Alvaro *et al.*, 2019 ▸); structural investigation of aperiodic crystalline materials (Hejny & Minkov, 2015 ▸, and literature therein); tracking and controlling chemical reactions in the solid state (Galica & Turowska-Tyrk, 2019 ▸; Sobczak *et al.*, 2021 ▸); and even tackling experimental charge density analysis for organic (Casati *et al.*, 2016 ▸) or inorganic (Gajda *et al.*, 2020 ▸) materials under pressure. In all the above applications, effective identification of systematic extinctions, access to high-resolution data and lack of superfluous features in Fourier maps (in other words, sufficient completeness of diffraction data) are necessary to identify new crystal phases and obtain unbiased results.

It is generally assumed that high completeness of diffraction data, comparable to those obtainable by means of standard crystallographic measurements, is unachievable in HP diffraction experiments. The exception is in a regular crystal system, where abundance of symmetry elements allows for the retrieval of almost all unique reflections. However, even if the symmetry of the analyzed sample is favorably high to begin with, a pressure-induced phase transition may lower it to the point where lack of completeness hampers further structural analysis.

The fact that convenient sample orientation can improve reciprocal space coverage and that tilting a crystal in a DAC and avoiding placement on its own crystal faces is beneficial are also generally acknowledged throughout the HP crystallographic community (Dziubek & Katrusiak, 2002 ▸; Dawson *et al.*, 2004 ▸). For instance, it is apparent from the optical images of loaded samples that the authors of the interesting work on ZIF-8 (Moggach *et al.*, 2009 ▸) put some effort into orienting their sample so that a vertex, and not a face, touches a diamond culet. However, the extent to which data completeness can be improved by effective sample orientation and how much can actually be gained depending on the sample Laue class has not been systematically investigated to date.

Our goal is to systematize and quantify the combined impact of radiation wavelength, DAC opening angle and, most importantly, the sample orientation on the achievable completeness of HP diffraction data. To that end, we present a comprehensive set of statistics describing data completeness in HP experiments, calculated using a series of numerical simulations performed with dedicated software. We show the extent to which experimental coverage can be improved by proper sample placement depending on the sample Laue class and utilized setup.

The viability of our approach is illustrated by the case of the luminescent 1,3-diacetylpyrene (2°AP) compound, which crystallizes as orthorhombic 2°AP-α under atmospheric conditions, but can undergo pressure-induced reversible single-crystal-to-single-crystal phase transitions, accompanied by symmetry lowering from the *mmm* to *mm*2 and eventually 2/*m* point group. The new polymorphs belong to the orthorhombic and monoclinic crystal systems, in which obtaining satisfactory data coverage poses a significant challenge and where proper sample orientation can have the greatest impact.

## 2. Completeness   

### 2.1. Definitions and methodology   

Volume *G* of International Tables of Crystallography (Hall *et al.*, 2006 ▸) defines completeness as a fraction of unique reflections measured to a given resolution, usually 0.6 Å^−1^ in non-macromolecular structures. While informative and concise, this definition leaves room for interpretation, as it does not strictly define a complete dataset. For example, a systemically extinct reflection is not considered missing, as its presence is unexpected in the first place.

Following this example, we can adapt the classical definition of completeness so that it disregards any reflections that are unobtainable using specific equipment, such as a DAC, similarly to how the classical definition disregards signals which are systematically extinct. This ‘applicable completeness’, 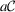 , is a ratio between numbers of *obtained* and *obtainable* unique reflections in a given space group and up to a certain resolution. According to this definition, 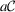 is a quality descriptor of experimental strategy and as such can always assume values between 0 and 1, independent of the equipment used. All experimental strategies can and should be planned, so that the applicable completeness of collected data is as close to 100% as possible.

The ratio between ‘classical’ completeness 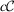 and ‘applicable’ completeness 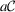 for any experiment compares the number of reflections *obtainable* using a given setup to the total count of signals available theoretically. As such, it is independent of individual measurement and describes the potency of experimental setup instead. This potency, 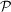 , is the maximum classical completeness of data collectable using a specified experimental setup.

For the majority of standard diffraction techniques, the availability of a modern goniometer and good incident sources rarely disallow the potency to reach 100%. Notable exceptions include diffraction techniques which utilize HP anvil cells or electron beam sources. In both cases, individual crystals can be rotated only partially during the experiment, preventing one from measuring diffraction from planes perpendicular to the incident beam.

### 2.2. Restrictions in HP experiments   

The subject of HP experiment potency was presented in a classical paper by Merrill & Bassett (1974 ▸). Therein, the authors present a formula for volume of reciprocal space accessible to their DAC for a monochromatic incident beam. Assuming a DAC opening angle of α and radiation wavelength of λ, it can be expressed as

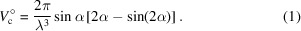

In typical experiments the diffraction is measured only up to a certain angle Θ, related to expected resolution *d*_min_ in accordance with Bragg’s Law. The region in which one collects data can be thus limited not only by the DAC, but also by a virtual sphere *S*_Θ_ of radius 2sinΘ/λ, bringing the accessible volume to

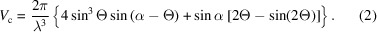

The percentage of reciprocal space accessible up to a given resolution threshold can be calculated analytically. It is the ratio between the HP-accessible volume *V*_c_ and the volume of space contained within the sphere *S*_Θ_, denoted *V*_Θ_: 

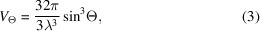




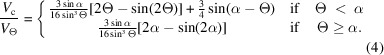

Typical values of the *V*_c_ to *V*_Θ_ ratio, presented in Table 1 ▸, approximate well the potency of the relevant HP experiment assuming all reflections are symmetry-independent. In reality, internal symmetry and sample orientation heavily influence the potency of the experiment, as information contained in unavailable reflections will be carried by their symmetry equivalents. Since the orientation of a single crystal relative to a DAC can not be changed during an XRD experiment, it will be considered a part of the experimental ‘setup’ (and not part of the measurement strategy) in further discussion.

**Figure 1:**
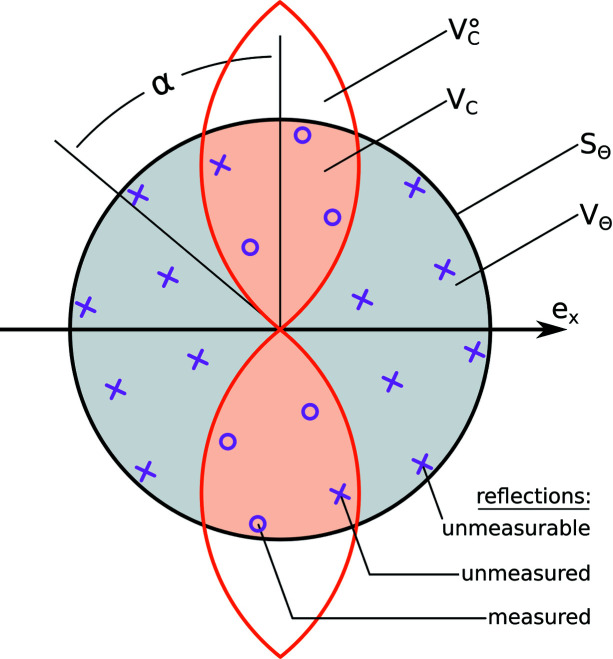
Total reciprocal space available to the pressure cell 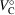 with the opening angle α, limited by investigated resolution sphere *S*_Θ_ down to *V*_C_. Assuming all reflections are symmetry-independent, the hypothetical experiment measured 6 out of 8 measurable and 20 total reflections, resulting in 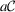 and 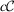 of 75 and 30%, respectively. 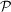 of this setup, assuming ‘orange’ orientation of the sample, is 40%.

The term ‘opening angle’ of a DAC is used somewhat inconsistently in the literature. Some authors define opening angle α as the angle between the vector normal to the diamond surface and a limiting vector which glides along the metal body of a DAC (Fig. 1 ▸), whereas others use it to describe the maximum value of 2θ obtainable in a diffraction experiment. In this work we use the term ‘opening angle’ to express the former, while terms ‘double opening angle’ or occasional ‘aperture’ relate to the latter.

### 2.3. Methods for estimating potency   

In simple cases, the potency of the experimental setup can be estimated analytically via direct integration over reciprocal space, as presented above. Unfortunately, derived formulas become exponentially more complicated with each symmetry element present; they are also not exact, as they describe the percentage of available reciprocal space volume instead of counting actual, symmetry-independent reflections.

**Figure 2:**
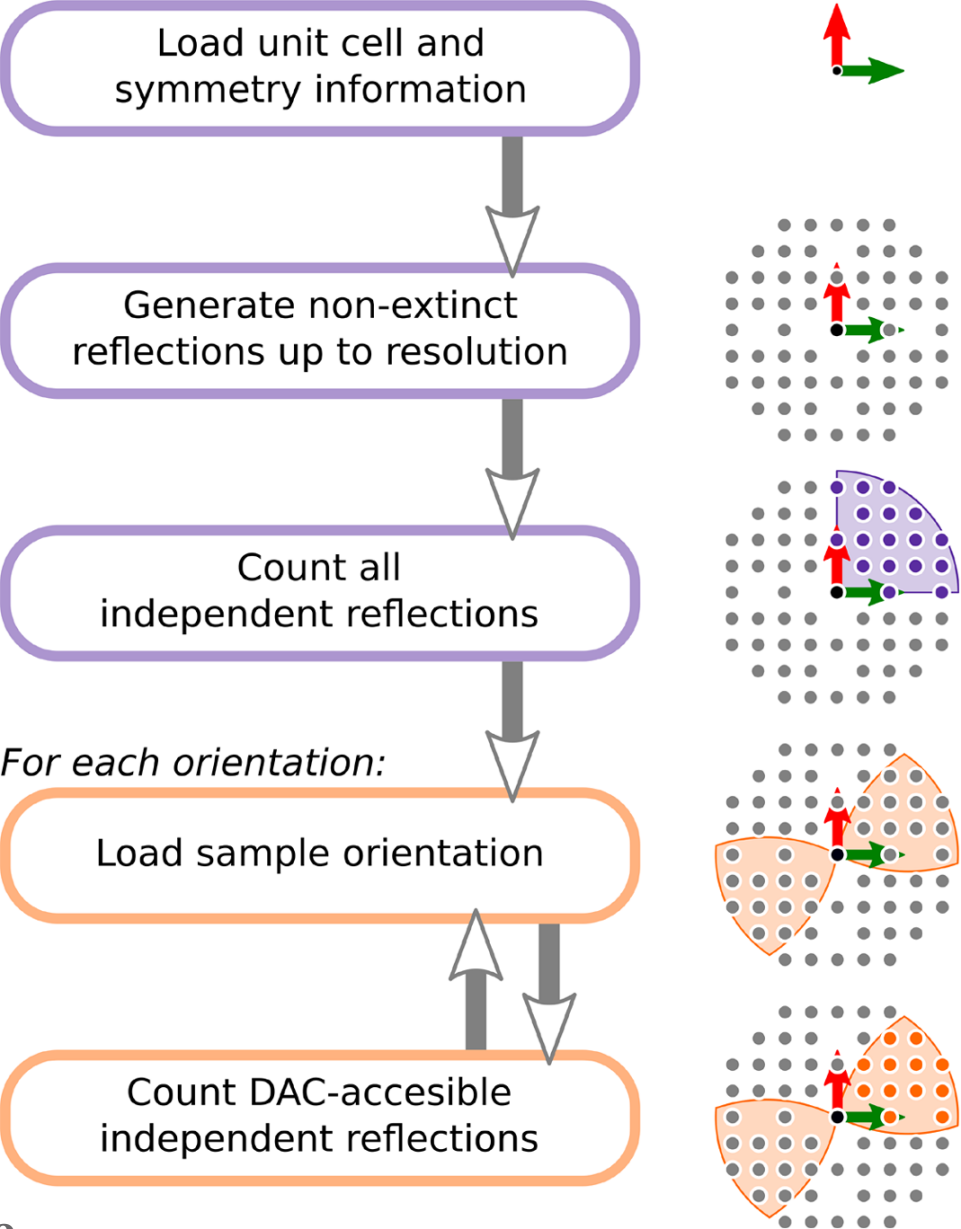
Procedure for potency evaluation using *Hikari*. In this example reciprocal space has *mm* point-group symmetry, there are 17 non-extinct symmetry-independent reflections (purple), 12 of which can be found within the DAC-accessible region (orange). Thus the potency of this experimental setup is ∼70.5%.

In order to calculate the impact of experimental conditions on potency, we instead opted for a direct numerical approach. For that reason a custom-made Python3 library nicknamed *Hikari* was utilized. The package has no dependency on other professional tools and instead operates using basic crystallographic formulae and generic functions from common libraries, making it very expandable, but relatively slow. The workflow used to estimate potency is presented in Fig. 2 ▸.

After interpreting input crystallographic data, *Hikari* generates reciprocal space nodes contained within a limiting sphere of desired radius. Extinct reflections are removed if necessary, after which all nodes are evaluated in terms of symmetry and independent nodes are counted. Then, for each investigated DAC orientation, an available region is determined, independent nodes within are counted and potency is evaluated.

The following procedure can be applied in many scenarios. Initial nodes can be generated based on an experimental diffraction file or only for a selected subspace (*i.e. hk0* plane). Resolution limits, wavelength and opening angle can be varied; any list of sample orientations can be used. Finally, various unit cells and symmetry settings can be investigated.

**Figure 3:**
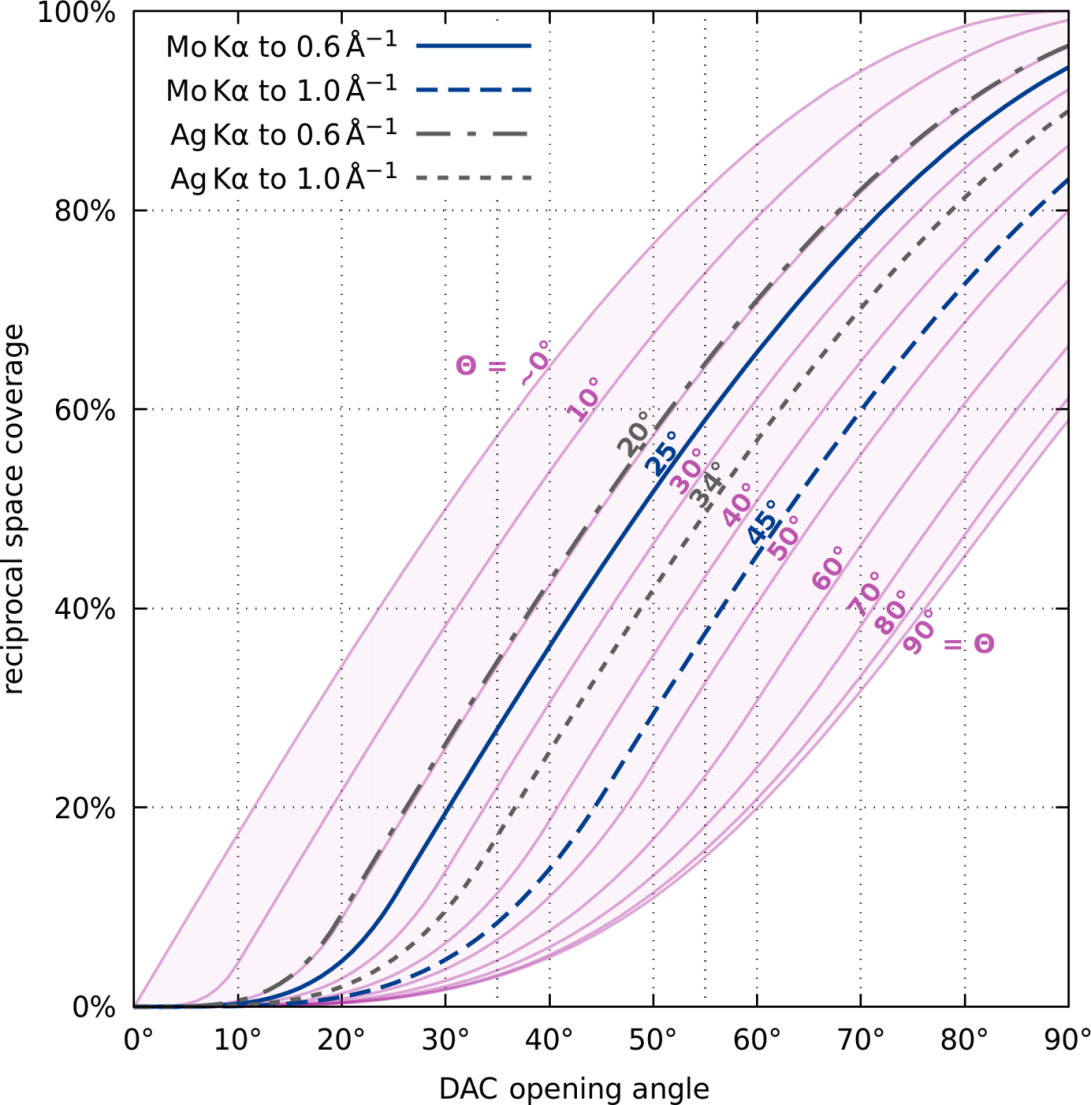
Reciprocal space coverage as a function of opening angle α presented for a series of Θ limits according to Equation (4). The Θ lines which translate into the most common combinations of radiation wavelengths and resolution limits have been highlighted. Assumed Laue class: −1.

The program can be used to evaluate existing experiment and experimental setup by counting symmetry-independent nodes excluded by a DAC (Table 2 ▸). Iterative application of this procedure for a series of orientations can be used to estimate optimal sample placement or investigate influence of DAC geometry (Figs. 3 ▸–7 and Section S5.1 of the supporting information). Feeding the algorithm with the correct structure factors allows one to estimate an impact of DAC restrictions on refinements against data collected previously (this functionality will be discussed elsewhere). In its current form, *Hikari* is available at https://github.com/Baharis/hikari. A dedicated server with interactive potency maps is under development.

### 2.4. Effect of opening angle   

The majority of single-crystal HP experiments are performed using the cell of Merrill-Bassett or a similar design (involving two opposite diamond anvils). Although individual implementations can vary, basic expressions for accessible volume, presented in Equation (4), tend to be fairly applicable. Thus, the DACs can be usually described using only their opening angle.

Apertures 2α of commercially available cells tends to vary between 60 and 80° (α = 30 or 40° accordingly). Quite recently, DACs with a nominal double opening angle of 120° (α = 60°) have also become available. In our experience the actual opening angle tends to be reduced due to gasket thickness or sample–gasket proximity (schematically explained in section S5.3 of the supporting information). For that reason, opening angles (α) of 35 or 55° will be discussed in more detail.

**Figure 4:**
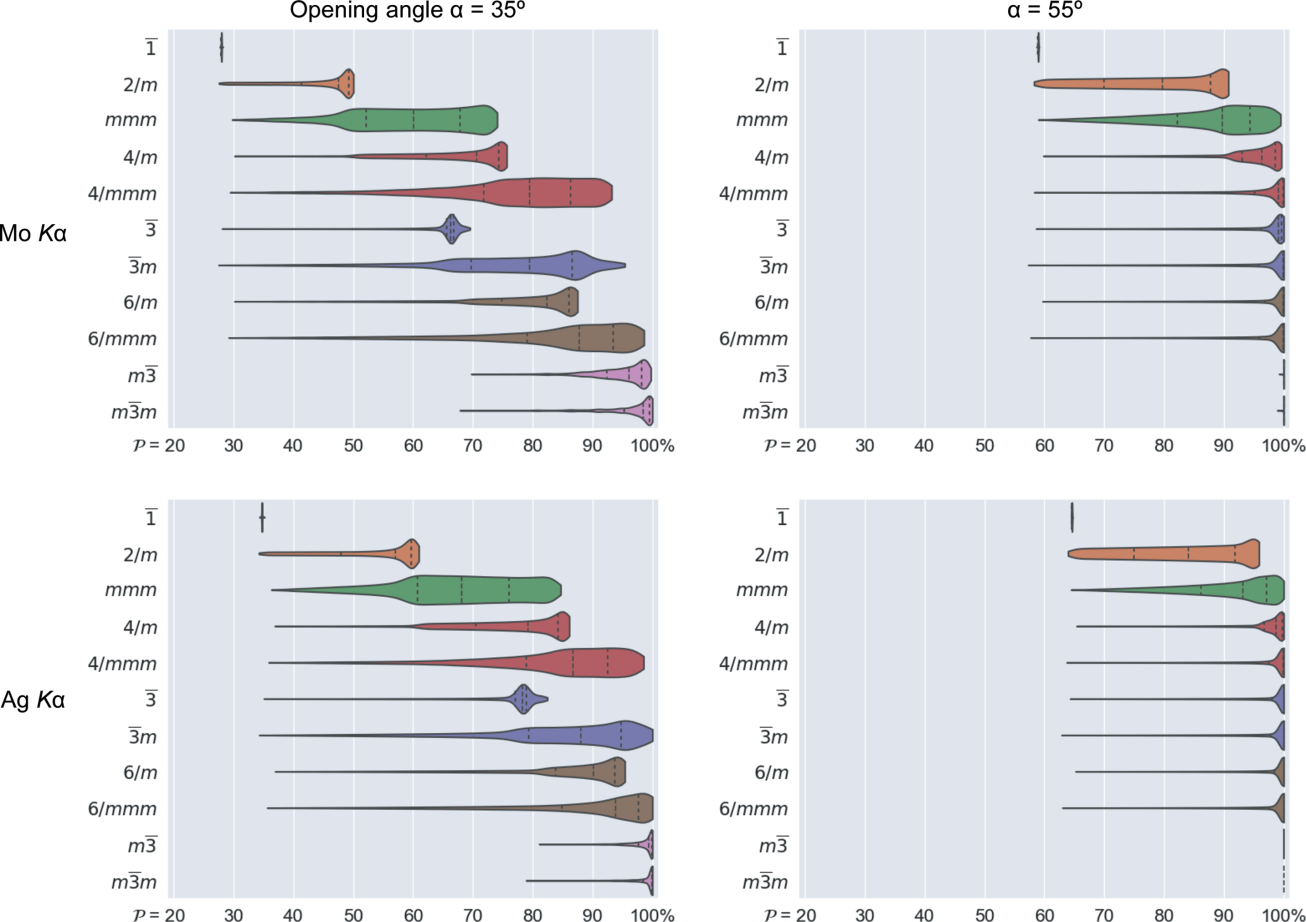
Violin plots representing the distribution of experiment potency for 50 000 uniformly distributed crystal orientations each, presented for commonly used laboratory wavelengths and DAC opening angles. The resolution limit for all plots assumed at sinΘ/λ = 0.6 Å^−1^. Dashed lines represent quartiles of each distribution. Further descriptive statistics are available in Table S5.1. Opening angle and sample orientation in conjunction with sample symmetry strongly influence potency even in the cubic system, where placing the crystal on one of the {100} planes can result in an incomplete dataset.

Reciprocal space coverage as a function of the opening angle of a DAC is graphically presented in Fig. 3 ▸. In the considered range of opening angles and resolution limits the coverage is almost proportional to Θ and the slopes of lines representing selected Θ values are very similar. The impact of the opening angle α is quite apparent. For the combination of Mo *K*α radiation and the IUCr diffraction limit of 0.6 Å^−1^, increasing the opening angle from 35 to 55° roughly doubles the reciprocal space coverage from 27 to 59%. The same effect is even more apparent in Fig. 4 ▸.

### 2.5. Effect of crystal orientation   

The internal symmetry and the placement of a crystal in the DAC play a secondary role when it comes to experimental potency, but their influence should not be underestimated. A DAC with a large aperture will always have an advantage over its counterparts, but attentive consideration of symmetry can increase the amount of collected data up to fourfold if performed correctly.

The disk-like shape of DAC-accessible volume features 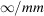 point group symmetry, which includes common symmetry elements of other point groups. In particular, the 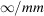 point group contains a center of inversion, which coincides with the appropriate center in crystal point groups at (000). Thus, lattice points (*hkl*) and ( *hkl* ) are always both available or both unavailable. Since the availability of space is centrosymmetric, the potency for each point group can be estimated using its Laue class, limiting the number of cases from 32 to 11.

The symmetry offered by point group −1 can not be used to increase the completeness of obtained data. Both symmetry elements present here coincide with the internal symmetry of the disk; as a result equivalent reflection pairs are always either both available or both unavailable, as discussed above. As such, in the case of Laue class −1 the potency is virtually independent of sample orientation and constitutes a baseline for other groups (Fig. 4 ▸). Nonetheless one can still attempt to use multiple differently oriented crystals to increase the final completeness (Casati *et al.*, 2016 ▸, 2017 ▸; Tchoń & Makal, 2019 ▸).

More possibilities are offered by the monoclinic crystal system, whose only Laue class 2/*m* is a subgroup of 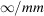 when the twofold axis coincides with either 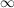 or an 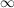 -perpendicular plane. In other cases it is possible for information outside the DAC-accessible disk to be included via registered, symmetry-equivalent reflection, effectively increasing the completeness of collected data above the value from Equation (4). Depending on the orientation, the potency of the HP experiment in the case of a monoclinic sample investigated using Mo *K*α radiation in a DAC with α = 35° up to sinθ/λ = 0.6 Å^−1^ can be as low as 27% or as high as 50% (Fig. 4 ▸, top left).

In a similar fashion the potency for samples whose diffraction patterns belong to the Laue class *mmm*, 3, 3 *m*, 4/*m*, 4/*mmm*, 6/*m* or 6/*mmm* can fall to the −1 baseline, as all those classes are sub-groups of 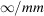 whenever 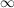 is parallel to their main axis. Ensuring that the main crystal axis does not coincide with the diamond culet normal has a great impact on final completeness, especially where α is small. In such case maximal achievable potency reaches 70% for trigonal 3, ∼75% for orthorhombic *mmm* or tetragonal 4/*m*, and over 85% for the remaining Laue classes (Mo *K*α up to sinθ/λ = 0.6 Å^−1^). Finally, while Laue classes *m* 3 and *m* 3 *m* are not subgroups of 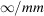 in any orientation, they still benefit from distancing their symmetry elements from axes and planes of 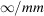 and experiment potency can be as low as 70% or as high as 100% (Fig. 4 ▸, top left).

**Figure 5:**
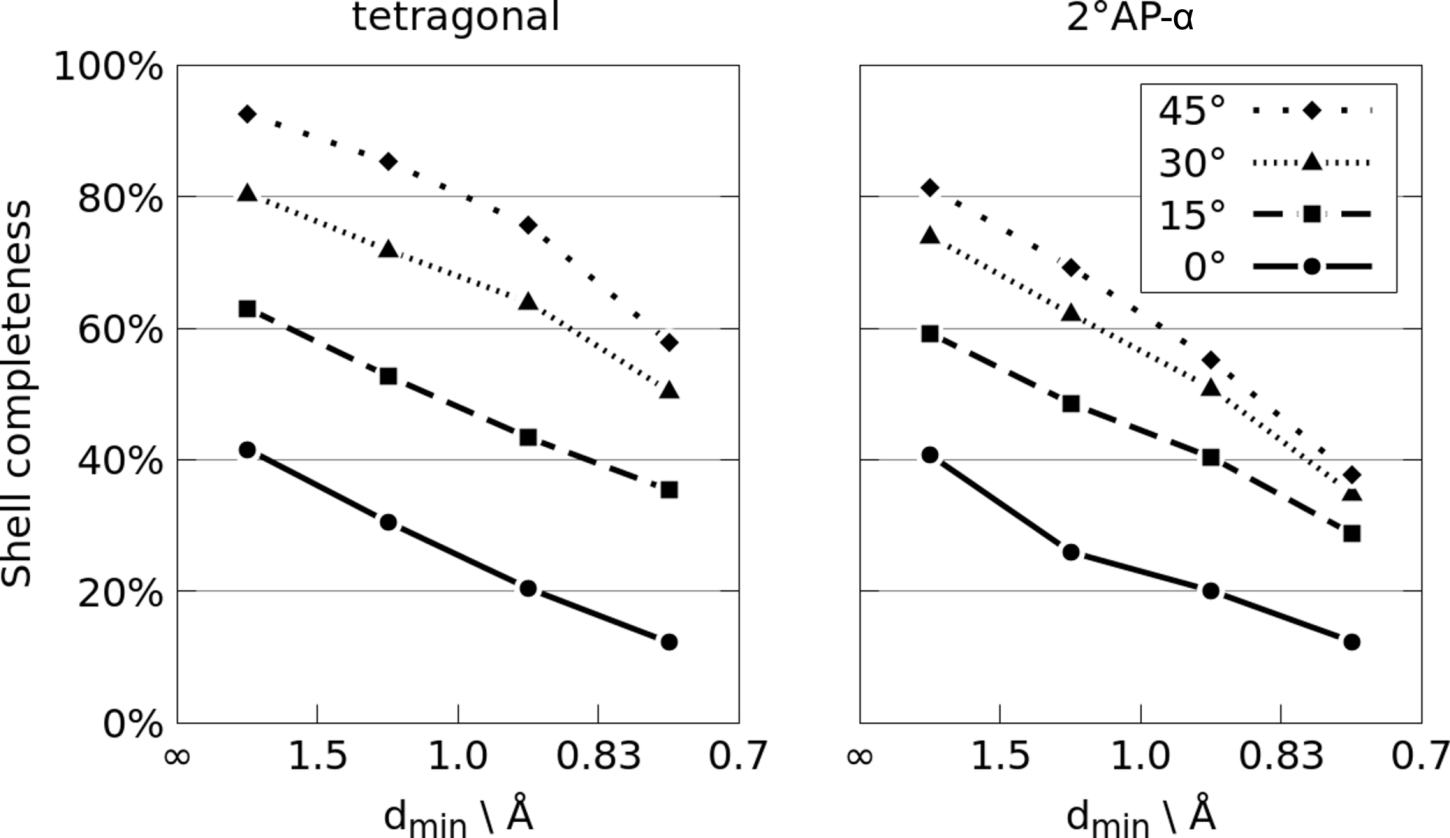
Average shell completeness for a model tetragonal crystal (left) and 2°AP-α (right) as a function of data resolution, assuming Mo *K*α wavelength and 35° opening angle. Different lines indicate topple angles between the (001) direction and the diamond culet normal. The relative increase in completeness is observed mainly at higher resolution, where data have high importance for correct space group determination. In the tetragonal case, tilting the crystal increases completeness fourfold in the highest resolution shell and ‘only’ about twofold in the lowest resolution shell.

It has been shown that various groups of reflections differ in their significance during crystal structure determination (Merli *et al.*, 2002 ▸). According to Parsons *et al.* (2012 ▸), weak high-resolution data can be very important in distinguishing between centrosymmetric and noncentrosymmetric models, as the leverage of weaker reflections seem to be higher during determination of their structure. Therein the leverage also did tend to increase with resolution. As shown in Fig. 5 ▸, toppling the main crystal axis with respect to the culet normal (*z* for tetragonal here) increases the relative completeness up to fourfold for the highest resolution shell and about twofold for the lowest resolution shell.

### 2.6. Orientation potency maps   

Since crystal placement has a significant impact on obtainable completeness, it would be of high interest to systematically present its influence on potency. The sample orientation, represented using a 3 × 3 **UB** matrix (Busing & Levy, 1967 ▸) defines the relation between the laboratory and sample reference systems as

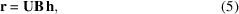

where **r** is expressed in the laboratory reference frame, while **h** contains Miller indices of the point in reciprocal space (Paciorek *et al.*, 1999 ▸).

In the laboratory reference frame, vector 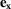 corresponds to the direction parallel to the incident beam which, in turn, coincides with the vector being normal to the diamond surface in a properly mounted anvil cell. As such, Miller indices **h** and Cartesian coordinates 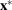 , describing the crystal face aligned with the diamond culet, can be obtained using Equations (6) and (7), respectively: 

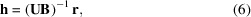




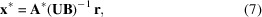

where 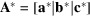 represents a transformation matrix between said reference frames. Just as vector 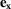 correlates to the rotational axis of the DAC in direct space, 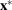 is aligned with the 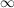 axis of the relevant DAC-accesible disk in reciprocal space. Note that, in the Ewald construction, vectors 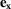 , 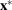 and **h** all have a common direction, but are expressed in different coordinate systems.

Due to the 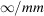 symmetry of the DAC-accessible region, its orientation in reciprocal space is unambiguously described using said vector 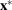 (or **h**). Accordingly, instead of investigating potency as a function of orientation, 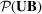 , it is sufficient to monitor the value of 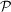 for each normalized vector 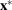 . This reduces the problem at hand to a simple 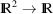 mapping, which can be represented in a comprehensible format.

**Figure 6:**
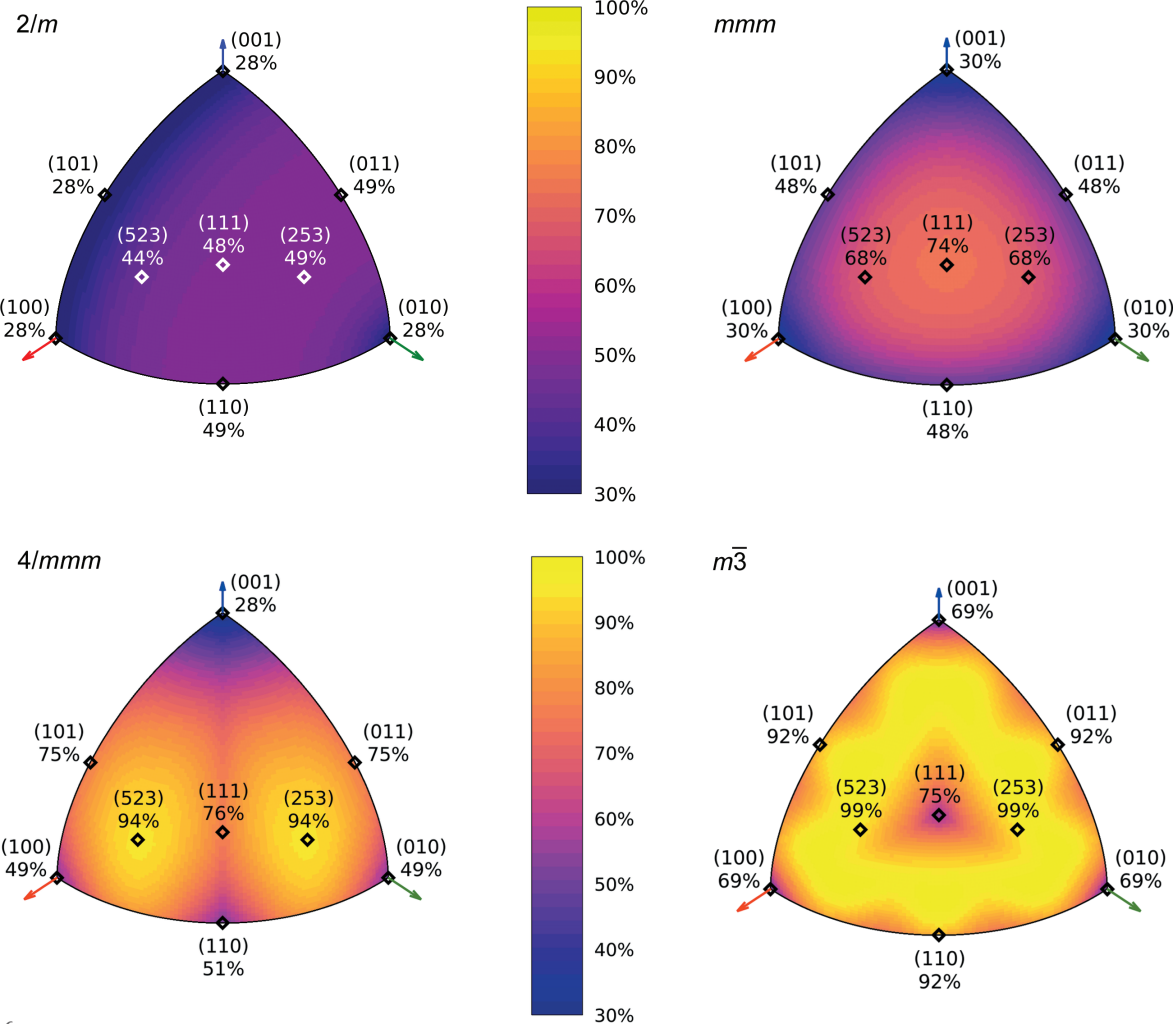
Potency heat maps for selected Laue classes, assuming the most common combination of α = 35° and resolution limit of sinΘ/λ = 0.6 Å^−1^. Unit-cell parameters for tetragonal: *a* = *b* = 15 Å, *c* = 20 Å; for other crystal systems: *a* = *b* = *c* = 20 Å. Potency as a function of sample orientation is mapped onto an octant of a unit sphere. Each point corresponds to normalized indices of a reciprocal space vector perpendicular to the DAC culet surface. Red, green and blue vectors represent x*, y* and z*, respectively. Examples of culet-perpendicular planes and resulting potency have been labeled and marked on the map using hollow points.

Since 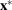 corresponds to a direction in three-dimensional space, it is natural to represent its functions on an unit sphere, akin to Kikuchi lines. Depending on the Laue class, usually only a fraction of this sphere is necessary. Potency as a function of 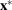 can be then represented using a color gradient, resulting in a figure analogous to a relief map of the Earth. Examples of such a potency map, generated for idealized samples, have been presented in Fig. 6 ▸ and more extensively in Section S5.1 of the supporting information.

Reading the potency map requires a basic understanding of relation between Miller indices **h** and reciprocal space coordinates 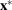 in the investigated crystal. For example, basis vectors 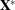 , 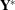 and 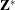 intersect a unit sphere at points 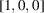 , 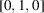 and 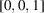 . Accordingly, the color of the sphere at their position describes the potency for the crystal placed on faces belonging to the family 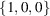 , 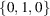 or 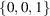 , respectively. However, the normalized vector 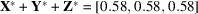 does not describe completeness for a crystal placed on the (111) face, but rather for the orientation featuring 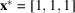 perpendicular to the diamond culet. In order to check the completeness for the crystal placed on the (*hkl*) face, one needs to revert from Miller indices **h** to Cartesian coordinates 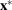 . Since Fig. 6 ▸ (bottom left) describes a tetragonal crystal with *a* = *b* = 15 Å, *c* = 20 Å, the potency of a crystal placed on the (111) face is visualized at the normalized vector 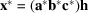 , or approximately [0.62, 0.62, 0.47], slightly below the center of the map.

The potency maps illustrate the impact of crystal symmetry on obtainable data completeness to the fullest extent and allow symmetry effects to be systematized and to expose certain paradoxes. Although most imaginable orientations in the case of *m* 3 symmetry in the cubic system promise data completeness of nearly 100%, there are still a few which can seriously compromise it. Most importantly, there is not a single best direction that would ensure satisfactory reciprocal space coverage for all crystal systems. Placing a crystal on the faces perpendicular to crystallographic directions should generally be avoided. However, placing an orthorhombic crystal on its own, well formed (111) face in a typical DAC (35° opening angle) warrants very high completeness up to ∼75% whereas the same maneuver reduces it for a cubic sample from an achievable 100% to ∼70% (Figs. 4 ▸ and 6 ▸). In essence, a well oriented orthorhombic crystal can yield data more complete than a very poorly oriented cubic one.

## 3. Application   

In order to present the applicability of completeness prediction and potency maps, 1,3-diacetylpyrene (2°AP, adapting the naming scheme from the first structural report by Rajagopal *et al.*, 2014 ▸) was selected as a test subject. The compound crystallizes in the space group *Pnma* under atmospheric conditions. A high variation of potency with crystal orientation in the orthorhombic system and the unique polymorphism of 2°AP make it a good subject for a feasibility study.

### 3.1. X-ray diffraction experiment   

2°AP was synthesized using the same synthesis protocol that has been formerly applied in obtaining 1-acetylpyrene and 1,8-diacetylpyrene. (Tchoń *et al.*, 2019 ▸, 2021 ▸). Recrystallization from a mixture of dichloromethane and *n*-pentane yielded mainly good-quality block crystals of known polymorph α, with traces of new monoclinic phase β, which will be discussed elsewhere.

XRD data were collected using the Rigaku Oxford Diffraction SuperNova four-circle diffractometer equipped with Eos CCD detector, molybdenum microsource (Mo *K*α, λ = 0.71073 Å) and an LN2 cooling device utilized while collecting a reference dataset at 100 K. Data collection and reduction were performed in *CrysAlisPRO* (Rigaku, 2015 ▸). Shape-based and empirical absorption correction were applied through the same software, using *SCALE3 ABSPack* (Oxford Diffraction 2015 ▸). Averaging, merging and outlier rejection were performed with *SORTAV* (Blessing, 1995 ▸). Further details of the procedures can be found in Tables S1.3 and S1.4 of the supporting information.

The reference and the three lowest-pressure datasets were solved in the space group *Pnma* using *SHELXT* and refined using *olex2.refine* (version 1.5-alpha) coupled with *NoSpherA2*, using the TAAM approach, with aspherical scattering factors provided by *DISCaMB* (Sheldrick, 2015*b* ▸; Bourhis *et al.*, 2015 ▸; Kleemiss *et al.*, 2021 ▸; Chodkiewicz *et al.*, 2018 ▸). The four higher-pressure datasets were solved and further refined assuming spherical scattering factors using *SHELXT*, *SHELXD* and *SHELXL* (Sheldrick, 2015*a* ▸,*b* ▸, 2010 ▸). Both procedures were performed within the *Olex2* GUI (Dolomanov *et al.*, 2009 ▸). Non-standard space group settings for new-found polymorphs, *Pn*2_1_ *a* for 2°AP-γ and *P*112_1_/*a* for 2°AP-δ, were chosen for consistency (see Table 3 ▸).

The exceptionally high completeness of the obtained data allowed all atoms to be easily found and refined without the need for restraints. The hydrogen atoms were constrained to their closest carbons and had their movement defined using the riding approximation in an independent atom model, but were refined freely in four *Pnma* datasets, including anisotropic displacement parameters at 5 kbar.

Refinement with an aspherical atom model could not be used for the highest-pressure 2°AP-δ phase as the algorithms could not properly handle the HKLF5 twinned data format. In the case of 2°AP-γ, the TAAM approach with unrestrained C—H distances did not improve refinement statistics and led to shortening of some C—H bond lengths. This was possibly the result of the coexistence of the traces of δ phase alongside the γ phase, contaminating the intensities of low-resolution reflections.

Molecular graphics were prepared using either* Olex2* (Dolomanov *et al.*, 2009 ▸) or *Mercury* software (version 4.3; Macrae *et al.*, 2008 ▸). The data were deposited with the Cambridge Structural Databaseand assigned CCDC deposition numbers 2096512–2096519.

### 3.2. Sample placement and data completeness   

The collection of reference data for 2°AP-α at 100 K required mounting a monocrystal on a Mitegen loop using a trace amount of paraton oil and running a series of ϕ-scans to collect all data up to sinθ/λ = 0.9 Å^−1^. The data collection strategy for the seven HP experiments, performed in three independent series, utilized the crystal mounting strategy proposed in this paper.

**Figure 7:**
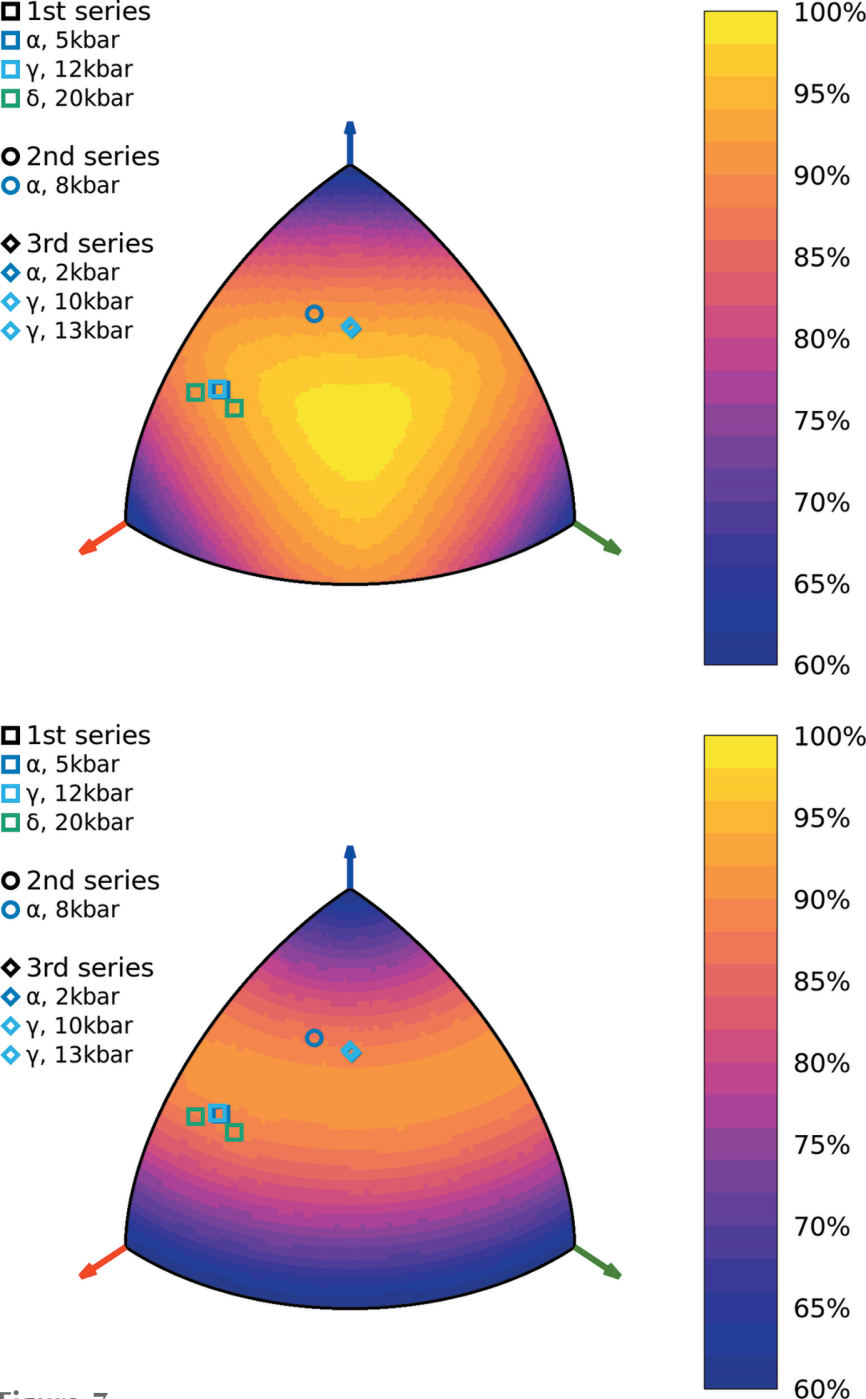
Potency heat maps prepared for 2°AP-α, 2°AP-γ and 2°AP-δ in the initial Laue class *mmm* (top), and in the final 2/*m* (bottom), assuming an opening angle of 55° and a 0.6 Å^−1^ resolution. The sample orientations in the HP experiments performed are marked; marked colors represent crystal phases and marked shapes correspond to individual packing series. As expected, the orientation is preserved in individual series, the only exception being δ, whose two domains appear during phase transition-induced twinning.

The orientation potency map prepared for 2°AP-α showed that the Mo *K*α wavelength and 55° opening angle could offer final completeness between 59 and 99%. Unfortunately, the lowest potency corresponded to placing the crystal on one of the {100} faces, both of which were among the largest in the specimen obtained. Meanwhile, the most potent geometry required the alignment of the diamond normal to 〈111〉 (or using Miller indices 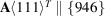 ), which did not correspond to any of the faces observed. Since the desired orientation could not be achieved naturally, in order to maximize potency of the prepared anvil cell, the specimen faces were indexed using preliminary XRD screening, after which they were mounted on the diamond culet with a trace amount of epoxy glue in an orientation as close as possible to that desired. The 2°AP-α potency map with final orientations marked is presented in Fig. 7 ▸.

After mounting the sample, a DACOne20 cell with an effective opening angle of 55° was equipped with a 300 µm steel gasket with a 300 µm hole, and filled with Paratone-N as a pressure-transferring medium. The pressure was estimated by the ruby fluorescence method using dedicated software. In total, seven collected datasets probed the pressure range up to 2 GPa. Their completeness was found to be very satisfactory as shown in Table 2 ▸.

### 3.3. Crystal packing   

2°AP-α and its HP relatives, γ and δ, exist as three variants of a common packing scheme. They arrange *a_z_* glide plane-related 2°AP molecules in offset β-type stacks (Desiraju & Gavezzotti, 1989 ▸) along **X**, forming jagged columns, a motif described previously by Rajagopal *et al.* (2014 ▸) as lamellar. These columns interlock with one another, creating a brick-layer motif analogous to those found in 1,8-diacetylpyrene.

**Figure 8:**
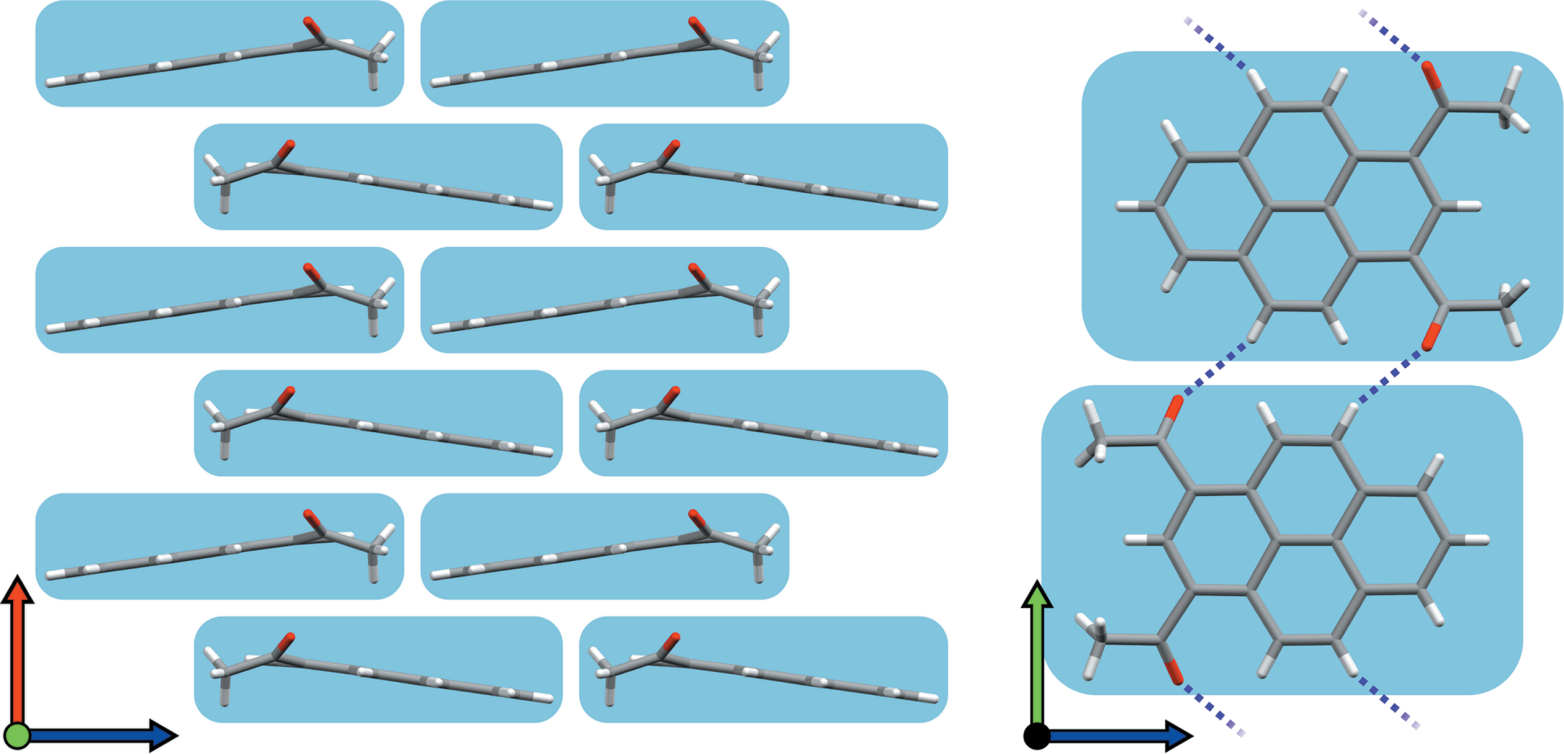
Crystal packing featured in the 2°AP-α family observed from *y* and *x*. Left: offset stacks of 2°AP molecules interlocked, creating brick-layer motifs in the *xz* plane. Right: neighboring brick-layers interact in the *y* direction via double hydrogen bonds on each side of each layer.

Just as π-stacking stabilizes the brickwork along *x* and *z*, polar interactions bind them along *y* (see Table S3.2). Each of the participating molecules donates a pyrene hydrogen and carbonyl oxygen atoms on both sides, allowing for two hydrogen bonds on each edge; thus infinite chains of intertwined 2°AP and 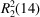 motifs (Etter, 1990 ▸) are formed in the process, as shown in Fig. 8 ▸.

**Figure 9:**
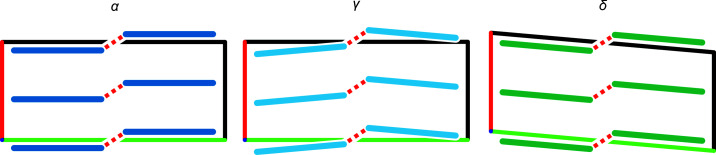
Schematic representation of crystal packing variations observed in 2°AP polymorphs as seen from *z*. Applied pressure gradually tilts individual stacks, leading to either orthorhombic γ or monoclinic δ.

Compression of 2°AP-α leads to a loss of symmetry, *e.g.* an increase of *Z*′ from 0.5 to 1 and the disappearance of the *m_y_* mirror plane. An additional degree of freedom granted to the *a_z_*-related molecules allows them to tilt around *z* which, in turn, causes the whole brickwork to lean in one direction. The tilt of closest layers might either alternate in a herringbone-like fashion or coincide. The first case preserves the 2_1_ axis in the *y* direction, resulting in a wavy-variant of α, orthorhombic 2°AP-γ. The second case preserves 2_1_ along *z*, but in turn topples the entire lattice to one side, producing monoclinic 2°AP-δ (Fig. 9 ▸). Due to equal probability of layers toppling ‘right’ and ‘left’, the crystal samples of δ tend to be twinned. Despite discarding half of symmetry elements, both γ and δ perfectly preserve the overall interaction network of 2°AP-α, as presented in Section S3 of the supporting information.

### 3.4. Phase transition   

New polymorphs of 2°AP discussed in this work are both obtained by the means of gradual pressurization of the commonly known crystal form α. On applying approximately 1 GPa of pressure, α undergoes a subtle phase transition towards orthorhombic 2°AP-γ. Both polymorphs feature virtually equal lattice constants and very similar packing schemes. The RMSD between molecular structures of α and γ investigated under similar pressure conditions is at the level of 0.02 Å.

**Figure 10:**
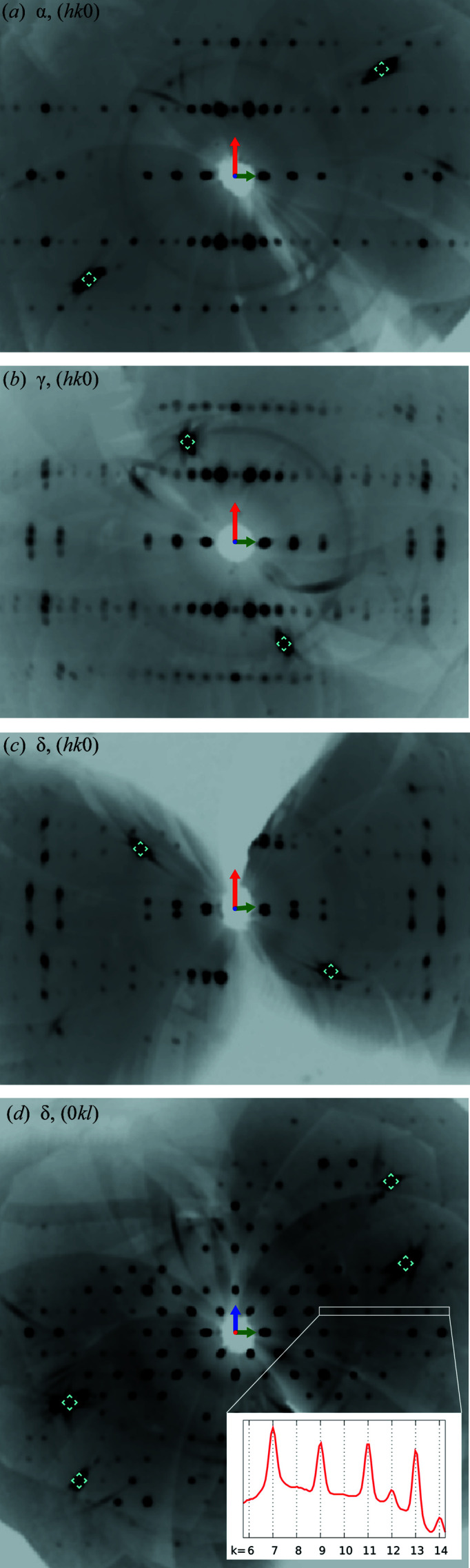
(*a*)–(*c*) Reconstructed diffraction patterns in (*hk*0) and (0*kl*) of α at 8 kbar, γ at 13 kbar and δ at 20 kbar, with diamond signals marked in cyan. Transition to a monoclinic system is gradual and already visible in the pattern of γ. (*d*) The δ (0*kl*) plane features partial extinctions characteristic for the *n_x_* glide plane; violations of such extinctions are only discernible at high resolution (inset, *k* = 12 and *k* = 14).

The crystal forms are even harder to distinguish in reciprocal space. 2°AP-α and 2°AP-γ share not only the same primitive orthorhombic unit cell, but also all extinction rules. Furthermore, structure factor statistics of 2°AP-γ show values characteristic for centrosymmetric systems despite *Pn*2_1_ *a* being a non-centrosymmetric space group. This may stem from the fact that the tilt oft the 2°AP molecule resulting in the loss of the *m_y_* mirror plane can occur in either direction, resulting in racemic twinning, as well as from the coexistence of the traces of δ alongside the γ phase, contaminating the intensities of low-resolution reflections, see Fig. 10 ▸(*b*). Finally, the prevalence of both space groups in the CSD is also rather similar. As such, differentiation between 2°AP-α and 2°AP-γ in reciprocal space was found to be extremely difficult. Instead, the correct space group was assigned based on agreement factors and the absence/presence of tilt in direct space (a detailed description is given in Section S1.6 of the supporting information). Although the difference between α and γ is very small, here it could be identified due to well defined atomic displacement parameters, resulting from high completeness of HP datasets collected.

The transition to the δ phase is, in turn, easy to observe due to the abrupt change of crystal system; however it is riddled with other problems. Firstly, the crystal samples of δ are systematically twinned around the 2_*y*_ axis (2_1*y*_ being lost in the transformation), in accordance with the former studies of twinning under pressure (Friese & Grzechnik, 2014 ▸). More interestingly, the transition does not occur at a fixed pressure. Traces of the δ diffraction pattern appear concurrently with γ, and then gradually overshadow it, dominating the image completely around 2 GPa. Within this range the reciprocal lattice tends to be decorated with three distinguishable patterns: one of disappearing γ and two for twinned δ.

Due to strong structural similarities the diffraction pattern of δ features almost the same extinctions as α and γ. The extinction rule of the *n_x_* glide plane, 0*kl*: *k*+*l* = 2*n*, is broken only for high-resolution reflections, as presented in Fig. 10(*d* ▸). This observation was possible due to the careful design of this experiment, as orienting the crystal on its largest face (001) or (001) would leave the (0*kl*) plane highly incomplete.

As far as our tests go, all phase transitions presented here are reversible, though some loss in overall pattern quality can be observed.

## 4. Conclusions   

The aim of this work was to systematize the combined impact of X-ray wavelength, DAC opening angle and the sample orientation, with the focus on the achievable completeness of HP diffraction data, and to quantify how much can the latter be improved by proper sample placement depending on the Laue class. This was accomplished in a series of numerical simulations performed with dedicated software. The resulting graphics – experiment potency maps – can be used as effective guides in designing HP diffraction experiments.

The usefulness of these guides has been tested in the case of luminescent 2°AP-α, undergoing reversible pressure-induced phase transitions from 2°AP-α (*Pnma*) to 2°AP-γ (*Pn*2_1_ *a*) and 2°AP-δ (*P*112_1_/*a*). The symmetry lowering occurring in those phase transitions could result in low data coverage and potentially hamper structural analysis. A combination of the wide DAC opening angle and a proper sample orientation has ensured well over 90% data coverage, *i.e.* completeness acceptable for conventional structural analysis, even for the monoclinic phase. This enabled unrestrained structure refinements for all phases, conducted with novel aspherical atomic scattering factors wherever possible. It allowed us to describe the mechanism governing these phase transitions and subsequent twinning, relying on a subtle tilt of the 2°AP molecule in the crystal lattice. Most importantly, it warranted careful examination of systematic extinction patterns owing to availability of highly complete reconstructions of (*hk*0), (*h*0*l*) and (0*kl*) reciprocal layers.

Unsurprisingly, the data presented confirm a great impact of the DAC opening angle, as changing it from 35 to 55° increases the minimum achievable coverage from ∼30 to ∼60% in all crystal systems. Compared with this aspect, choosing a shorter X-ray wavelength has very little influence and may have additional drawbacks in beam intensity loss, unless very enhanced X-ray optics or synchrotron sources are available. The most important general observation, however, is that sample pre-orientation within a DAC has a profound impact on data completeness. Given a certain laboratory X-ray source and a DAC, sample placement in a DAC is the only variable over which a researcher exercises control and which can be widely varied. For instance, for monoclinic samples, using Mo *K*α radiation and a DAC with a typical 35° opening angle completeness of ∼50% can be reached consistently up to the IUCr resolution limit by avoiding placing the sample on the (010) face. Meanwhile it is widely accepted that a completeness of 30% is typical and 40% is relatively high for this system. In the case of the 4/*mmm* Laue class in the tetragonal system, a sample tilt by only around 15°, easily achievable with a tiny prop from expoy resin, can increase experiment potency from 50 to 80% (94% being the maximum). In particular, a well oriented orthorhombic crystal can yield data completeness higher than a poorly oriented cubic one. This indicates that even the most coverage-demanding HP studies can be successfully conducted under laboratory conditions for samples that are commonly deemed of too low-symmetry, once the experiment is carefully planned and a crystal properly oriented. While pre-orientation will be impossible for pressurizing liquids and ineffective for triclinic samples, it can substantially improve completeness and hence data quality for a wide variety of investigated systems.

Although the usefulness of sample tilting in a DAC and of avoiding placement on its own crystal faces is generally accepted in the community, this work, for the first time, quantifies exactly how much can be gained by this intuitive approach. It also provides guides which should enable users to plan their experiments with the sample and equipment at hand. The guides presented also allow us to avoid certain simplifications and pitfalls. For instance, placing an orthorhombic crystal on its own, well formed (111) face in a typical DAC with a 35° opening angle warrants very high completeness up to ∼75%, whereas the same maneuver reduces completeness for a cubic sample from an achievable 100% down to ∼70%.

The impact of specimen symmetry on coverage during data collection has been addressed to some extent in the highly acclaimed work by Casati *et al.* (2017 ▸). The authors presented ‘expected data coverage for data collection in a DAC with an (double) opening of 80°’. According to one of the authors, the orientation of simulated samples was selected arbitrarily, aimed at high completeness, with special attention being paid to the investigation of axes and planes where systematic extinctions might occur. Simulations performed in the current work suggested that those arbitrary orientations were in fact unfavorable in considered Laue classes and, as such, not representative. Contrary to arbitrary tests, our results are comprehensive and applicable to a wide range of experimental scenarios.

## 5. Related literature   

The following references are cited in the supporting information: Dovesi *et al.* (2018 ▸); Erba *et al.* (2014 ▸); González (2010 ▸); Groom *et al.* (2016 ▸); Turner *et al.* (2014 ▸, 2017 ▸); Vinet *et al.* (1987 ▸). 

## Supplementary Material

Crystal structure: contains datablock(s) 2oAPal_0kbar, 2oAPal_2kbar, 2oAPal_5kbar, 2oAPal_8kbar, 2oAPga_10kbar, 2oAPga_12kbar, 2oAPga_13kbar, 2oAPde_20kbar. DOI: 10.1107/S2052252521009532/fc5056sup1.cif

Supporting figures and tables. DOI: 10.1107/S2052252521009532/fc5056sup2.pdf

CCDC references: 2096512, 2096513, 2096514, 2096515, 2096516, 2096517, 2096518, 2096519

## Figures and Tables

**Table 1:** Percentage of reciprocal space available in the HP experiment calculated analytically based on Equation (4), assuming a DAC double opening angle 2α of 70°

Resolution	*d*_min_ = 1.50 Å	*d*_min_ = 0.83 Å	*d*_min_ = 0.50 Å
λ = 1.54187 Å	20.99%	3.79%	0.83%
λ = 0.71069 Å	41.83%	27.79%	8.45%
λ = 0.5608 Å	45.27%	34.60%	17.15%

**Table 2:** Classical completeness, applicable completeness and potency of the data collected to 0.6 Å^−1^ in the HP experiments performed

Series	Phase, pressure (kbar)	c{\cal C}	a{\cal C}	{\cal P}
Third	α, 2	92.91%	99.49%	93.39%
First	α, 5	91.91%	98.51%	93.30%
Second	α, 8	91.12%	99.64%	91.45%
Third	γ, 10	93.81%	100.00%†	93.81%
First	γ, 12	92.14%	98.68%	93.37%
Third	γ, 13	93.64%	100.19%†	93.46%
First	δ, 20	92.91%	99.66%	93.22%

† Artificially inflated by weak signals of δ erroneously included by software.

**Table 3:** Basic crystallographic data of the investigated 2°AP polymorphs

Phase	α	α	α	α	γ	γ	γ	δ
Pressure (kbar)	0	2	5	8	10	12	13	20
Space group	*Pnma*	*Pnma*	*Pnma*	*Pnma*	*Pn*2_1_ *a*	*Pn*2_1_ *a*	*Pn*2_1_ *a*	*P*112_1_/*a*
*a* (Å)	7.21024 (18)	7.2462 (11)	7.150 (10)	7.0642 (11)	7.0300 (9)	7.0202 (15)	6.9714 (16)	6.8697 (19)
*b* (Å)	16.4876 (5)	16.536 (3)	16.17 (3)	16.220 (2)	16.147 (2)	16.0775 (18)	16.139 (5)	15.960 (2)
*c* (Å)	11.2792 (3)	11.255 (3)	11.288 (9)	11.166 (2)	11.1214 (19)	11.1610 (16)	11.072 (3)	11.1099 (18)
γ (°)	90	90	90	90	90	90	90	95.074 (17)
*R*_int_ (%)	0.095	0.157	0.072	0.109	0.103	0.125	0.141	0.153
*R*_1_ (*I* > 2σ) (%)	0.037	0.055	0.034	0.056	0.065	0.075	0.089	0.081
