# Supplementary material for: Maximizing completeness in single-crystal high-pressure diffraction experiments: phase transitions in 2°AP
Source: IUCrJ. 2021 Oct 15;8(Pt 6):1006–17. doi: 10.1107/S2052252521009532 (PMC8562673; doi:10.1107/S2052252521009532)
Supplement: Supplementary file 2 [file m-08-01006-sup2.pdf]

**IUCrJ**

**Volume 8 (2021)**

**Supporting information for article:**

# Maximizing completeness in single-crystal high-pressure diffraction experiments: phase transitions in 2oAP

**D. Tchoń and A. Makal**

# Maximizing completeness in single-crystal high-pressure diffraction experiments: phase transitions in 2°AP

Daniel Tchoń and Anna Makal,
contact: *amakal@chem.uw.edu.pl*

October 11, 2021

# Contents

**S1 Methods** **S3**
S1.1 X-ray data collection . . . . . S3
S1.2 Space group choice . . . . . S9
S1.3 Deposition . . . . . S12

**S2 Structure description** **S13**
S2.1 Phase transitions . . . . . S13

**S3 Lattice interactions** **S14**

**S4 Theoretical calculations** **S16**

**S5 Completeness** **S19**
S5.1 Generating potency maps . . . . . S19
S5.2 Reading potency maps . . . . . S29
S5.3 Opening angle . . . . . S30

**S6 Bibliography** **S31**

# Chapter S1

# Methods

## S1.1 X-ray data collection

Crystals used in the diffraction experiments were primarily obtained by recrystallization from dichloromethane and $n$-pentane mixture, yielding clear large yellow prism-like blocks, with well-formed faces (see Figure S1.2).

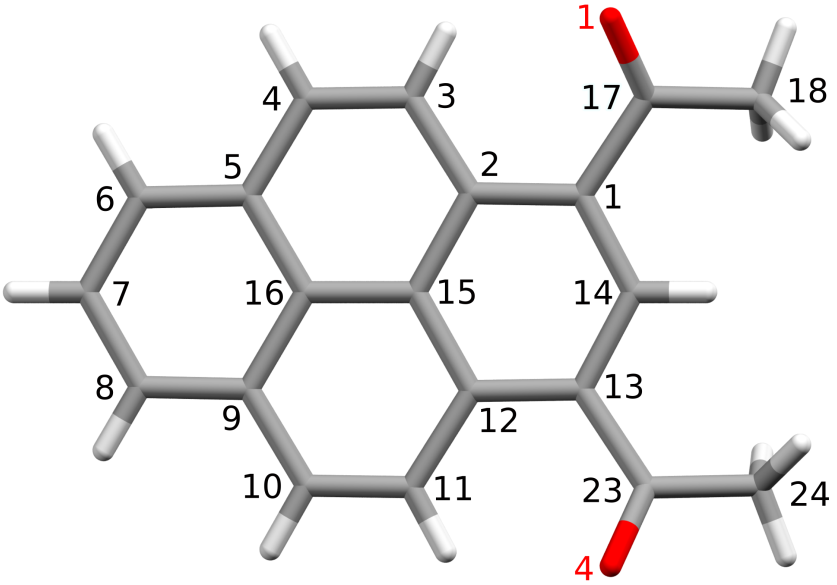


***Figure S1.1:*** *Labeling scheme of 2°AP molecule. Hydrogen atoms inherit label after closest carbon atom. Due to a presence of additional mirror plane, polymorph α utilizes only labels from top half of the Figure (O1, C1–7, C14–18).*

***Table S1.1:*** *Refined component ratios, number of isolated and overlapping reflections, and twinning matrix for diffraction experiment performed on 2°AP-δ in 20kbar. The second matrix corresponds to rotation by 180° around vector [1.00 0.00 -0.04] in reciprocal space or vector [1.00 0.00 0.01] in direct space, as given by CrysAlisPro.*

| Twin | Refined component ratio | Isolated reflections | Overlapped reflections | Twinning matrix |
|---|---|---|---|---|
| 1 | 0.5769 | 9604 | 10301 | $\begin{bmatrix} 1.0000 & 0.0000 & 0.0000 \\ 0.0000 & 1.0000 & 0.0000 \\ 0.0000 & 0.0000 & 1.0000 \end{bmatrix}$ |
| 2 | 0.4231 | 9494 | 10301 | $\begin{bmatrix} 0.9990 & -0.0027 & 0.0098 \\ -0.0002 & -0.9984 & -0.0008 \\ -0.0749 & 0.0002 & -1.0000 \end{bmatrix}$ |

***Table S1.2:*** *Orientation matrices and vectors perpendicular to diamond surface (expressed in Cartesian* $\mathbf{x}^*$ *and fractional* $\mathbf{h}^*$ *coordinates) listed for all crystal samples utilized in performed diffraction experiments.*

| Experiment | Orientation matrix | DAC-perpendicular vector |
|---|---|---|
| | —SERIES I— | |
| 2°AP-$\alpha$, 5kbar | $\begin{bmatrix} -0.07635 & -0.00835 & 0.03859 \\ -0.01825 & -0.03679 & -0.03154 \\ 0.06093 & -0.02151 & 0.03894 \end{bmatrix}$ | $\mathbf{x}^* = \begin{bmatrix} -0.76945 & -0.19523 & 0.60813 \end{bmatrix}$ <br> $\mathbf{h}^* = \begin{bmatrix} -0.58863 & -0.33777 & 0.73446 \end{bmatrix}$ |
| 2°AP-$\gamma$, 12kbar | $\begin{bmatrix} -0.07823 & 0.00800 & -0.03858 \\ -0.01892 & 0.03742 & 0.03142 \\ 0.06114 & 0.02192 & -0.03954 \end{bmatrix}$ | $\mathbf{x}^* = \begin{bmatrix} -0.77375 & 0.18281 & -0.60654 \end{bmatrix}$ <br> $\mathbf{h}^* = \begin{bmatrix} -0.59277 & 0.32075 & -0.73875 \end{bmatrix}$ |
| 2°AP-$\delta$, 20kbar, twin I | $\begin{bmatrix} -0.08263 & 0.00536 & -0.03667 \\ -0.01671 & 0.03679 & 0.03238 \\ 0.06030 & 0.02438 & -0.04088 \end{bmatrix}$ | $\mathbf{x}^* = \begin{bmatrix} -0.81009 & 0.12183 & -0.57351 \end{bmatrix}$ <br> $\mathbf{h}^* = \begin{bmatrix} -0.61812 & 0.34213 & -0.70772 \end{bmatrix}$ |
| 2°AP-$\delta$, 20kbar, twin II | $\begin{bmatrix} 0.08251 & 0.01162 & 0.03675 \\ 0.01682 & 0.03807 & -0.03236 \\ -0.06016 & 0.01982 & 0.04088 \end{bmatrix}$ | $\mathbf{x}^* = \begin{bmatrix} 0.77660 & 0.26123 & 0.57328 \end{bmatrix}$ <br> $\mathbf{h}^* = \begin{bmatrix} 0.60264 & 0.34529 & 0.71945 \end{bmatrix}$ |
| | —SERIES II— | |
| 2°AP-$\alpha$, 8kbar | $\begin{bmatrix} -0.04669 & -0.01341 & -0.05278 \\ -0.06775 & 0.03182 & 0.00693 \\ 0.05770 & 0.02683 & -0.03458 \end{bmatrix}$ | $\mathbf{x}^* = \begin{bmatrix} -0.46313 & -0.30474 & -0.83225 \end{bmatrix}$ <br> $\mathbf{h}^* = \begin{bmatrix} -0.29682 & -0.44844 & -0.84309 \end{bmatrix}$ |
| | —SERIES III— | |
| 2°AP-$\alpha$, 2kbar | $\begin{bmatrix} -0.04052 & -0.01815 & 0.05057 \\ 0.08389 & 0.00521 & 0.03170 \\ -0.03042 & 0.03859 & 0.01964 \end{bmatrix}$ | $\mathbf{x}^* = \begin{bmatrix} -0.41358 & -0.42230 & 0.80661 \end{bmatrix}$ <br> $\mathbf{h}^* = \begin{bmatrix} -0.25313 & -0.58984 & 0.76682 \end{bmatrix}$ |
| 2°AP-$\gamma$, 10kbar | $\begin{bmatrix} 0.04235 & 0.01869 & 0.05118 \\ -0.08640 & -0.00538 & 0.03212 \\ 0.03067 & -0.03949 & 0.01982 \end{bmatrix}$ | $\mathbf{x}^* = \begin{bmatrix} 0.41483 & 0.41941 & 0.80747 \end{bmatrix}$ <br> $\mathbf{h}^* = \begin{bmatrix} 0.25098 & 0.58284 & 0.77286 \end{bmatrix}$ |
| 2°AP-$\gamma$, 13kbar | $\begin{bmatrix} 0.04276 & 0.01883 & 0.05146 \\ -0.08711 & -0.00581 & 0.03217 \\ 0.03062 & -0.03956 & 0.01943 \end{bmatrix}$ | $\mathbf{x}^* = \begin{bmatrix} 0.41289 & 0.41169 & 0.81242 \end{bmatrix}$ <br> $\mathbf{h}^* = \begin{bmatrix} 0.24927 & 0.57539 & 0.77897 \end{bmatrix}$ |

*Table S1.3: Data reduction and refinement details for 2°AP-α.*

| | | | | |
|---|---|---|---|---|
| **Crystal data** | | | | |
| Sum formula, Mr | $C_{20}H_{14}O_2$, 286.31 | | | |
| Crystal system | orthorhombic | | | |
| Symmetry information | $Pnma$, Z=4, Z'=0.5 | | | |
| Crystal phase | $\alpha$ | $\alpha$ | $\alpha$ | $\alpha$ |
| Pressure \ GPa | 0.00 | 0.25(10) | 0.52(10) | 0.78(10) |
| $a$ \ Å | 7.21024(18) | 7.2462(11) | 7.150(10) | 7.0642(11) |
| $b$ \ Å | 16.4876(5) | 16.536(3) | 16.17(3) | 16.220(2) |
| $c$ \ Å | 11.2792(3) | 11.255(3) | 11.288(9) | 11.166(2) |
| $\gamma$ \ ° | 90 | 90 | 90 | 90 |
| $V$ \ Å$^3$ | 1340.86(6) | 1348.6(4) | 1305(3) | 1279.4(4) |
| Density \ g cm$^{-3}$ | 1.418 | 1.410 | 1.457 | 1.486 |
| **Data collection** | | | | |
| Temperature | 100.00(11) | 293.4(5) | 295.4(6) | 289(10) |
| Radiation type | micro-focus sealed X-ray tube | | | |
| Wavelength \ Å | 0.71073 | 0.71073 | 0.71073 | 0.71073 |
| Absorption correction | Empirical multi-scan (SCALE3 ABSPACK) | | | |
| $\mu$ \ $mm^{-1}$ | 0.091 | 0.090 | 0.093 | 0.095 |
| Tmin | 0.816 | 0.01456 | 0.26651 | 0.07503 |
| Tmax | 1.000 | 1.00000 | 1.00000 | 1.00000 |
| Diffractometer | Rigaku Oxford Diffraction SuperNova | | | |
| Resolution \ Å | 0.5509 | 0.7248 | 0.7390 | 0.7392 |
| Reflections: measured | 77511 | 18918 | 13312 | 23014 |
| independent | 4492 | 1577 | 1409 | 1436 |
| observed | 2997 | 853 | 845 | 804 |
| h range | $[-13, 13]$ | $[-9, 9]$ | $[-8, 8]$ | $[-9, 9]$ |
| k range | $[-30, 30]$ | $[-19, 22]$ | $[-21, 21]$ | $[-21, 21]$ |
| l range | $[-20, 20]$ | $[-13, 13]$ | $[-13, 12]$ | $[-13, 12]$ |
| Rint | 0.0949 | 0.1567 | 0.0719 | 0.1095 |
| Rsigma | 0.0620 | 0.1159 | 0.0727 | 0.0971 |
| Completeness to 0.83Å | 99.8% | 92.8% | 91.8% | 90.5% |
| **Refinement** | | | | |
| R1 | 0.0759 | 0.1329 | 0.0932 | 0.1350 |
| R1 ($I > 2\sigma$) | 0.0374 | 0.0547 | 0.0341 | 0.0561 |
| wR2 | 0.0616 | 0.1352 | 0.0602 | 0.1529 |
| wR2 ($I > 2\sigma$) | 0.0554 | 0.1080 | 0.0487 | 0.1229 |
| GooF | 0.9394 | 1.0221 | 0.9283 | 1.0348 |
| No. of parameters | 172 | 129 | 173 | 129 |
| No. of restraints | 0 | 0 | 0 | 0 |
| $\Delta\rho_{max}$ \ $e$Å$^{-3}$ | 0.4887 | 0.5950 | 0.3988 | 0.5899 |
| $\Delta\rho_{min}$ \ $e$Å$^{-3}$ | -0.5380 | -0.7652 | -0.3837 | -0.5589 |
| $\Delta\rho_{rms}$ \ $e$Å$^{-3}$ | 0.1086 | 0.1352 | 0.0951 | 0.1343 |

***Table S1.4:*** *Data reduction and refinement details for 2°AP-$\gamma$ and 2°AP-$\delta$.*

| | | | | |
|---|---|---|---|---|
| **Crystal data** | | | | |
| Sum formula, Mr | $C_{20}H_{14}O_2$, 286.31 | | | |
| Crystal system | orthorhombic | | | monoclinic |
| Symmetry information | $Pn2_1a$, Z=4, Z'=1 | | $P112_1/a$, Z=4, Z'=1 | |
| Crystal phase | $\gamma$ | $\gamma$ | $\gamma$ | $\delta$ |
| Pressure \ GPa | 0.97(10) | 1.16(10) | 1.30(10) | 2.03(10) |
| $a$ \ Å | 7.0300(9) | 7.0202(15) | 6.9714(16) | 6.8697(19) |
| $b$ \ Å | 16.147(2) | 16.0775(18) | 16.139(5) | 15.960(2) |
| $c$ \ Å | 11.1214(19) | 11.1610(16) | 11.072(3) | 11.1099(18) |
| $\gamma$ \ ° | 90 | 90 | 90 | 95.074(17) |
| $V$ \ Å$^3$ | 1262.4(3) | 1259.7(4) | 1245.7(6) | 1205.8(15) |
| Density \ g cm$^{-3}$ | 1.506 | 1.510 | 1.527 | 1.567 |
| | | | | |
| **Data collection** | | | | |
| Temperature | 293(2) | 295.4(9) | 294(2) | 295.4(4) |
| Radiation type | micro-focus sealed X-ray tube | | | |
| Wavelength \ Å | 0.71073 | 0.71073 | 0.71073 | 0.71073 |
| Absorption correction | Empirical multi-scan (SCALE3 ABSPACK) | | | |
| $\mu$ \ $mm^{-1}$ | 0.096 | 0.096 | 0.098 | 0.100 |
| Tmin | 0.06286 | 0.08017 | 0.35833 | 0.50558 |
| Tmax | 1.00000 | 1.00000 | 1.00000 | 1.00000 |
| Diffractometer | Rigaku Oxford Diffraction SuperNova | | | |
| Resolution \ Å | 0.7170 | 0.7400 | 0.7134 | 0.7356 |
| Reflections: measured | 32750 | 20057 | 29293 | 28843 |
| independent | 3030 | 2639 | 2962 | 4304 |
| observed | 1829 | 1520 | 1503 | 1532 |
| h range | $[-9,9]$ | $[-8,7]$ | $[-9,9]$ | $[-8,8]$ |
| k range | $[-21,21]$ | $[-14,14]$ | $[-13,13]$ | $[-21,21]$ |
| l range | $[-13,13]$ | $[-21,20]$ | $[-21,21]$ | $[-14,14]$ |
| Rint | 0.1033 | 0.1248 | 0.1402 | 0.1529 |
| Rsigma | 0.1039 | 0.1233 | 0.1468 | 0.2781 |
| Completeness to 0.83Å | 93.4% | 93.0% | 93.3% | 92.5% |
| | | | | |
| **Refinement** | | | | |
| R1 | 0.1323 | 0.1502 | 0.1895 | 0.2303 |
| R1 ($I > 2\sigma$) | 0.0652 | 0.0753 | 0.0895 | 0.0813 |
| wR2 | 0.1616 | 0.1997 | 0.2627 | 0.1843 |
| wR2 ($I > 2\sigma$) | 0.1388 | 0.1626 | 0.2190 | 0.1556 |
| GooF | 1.022 | 0.992 | 1.007 | 0.868 |
| No. of parameters | 202 | 202 | 202 | 203 |
| No. of restraints | 1 | 1 | 1 | 0 |
| $\Delta\rho_{max}$ \ $e$Å$^{-3}$ | 0.245 | 0.276 | 0.347 | 0.282 |
| $\Delta\rho_{min}$ \ $e$Å$^{-3}$ | -0.259 | -0.339 | -0.339 | -0.297 |
| $\Delta\rho_{rms}$ \ $e$Å$^{-3}$ | 0.058 | 0.076 | 0.072 | 0.074 |

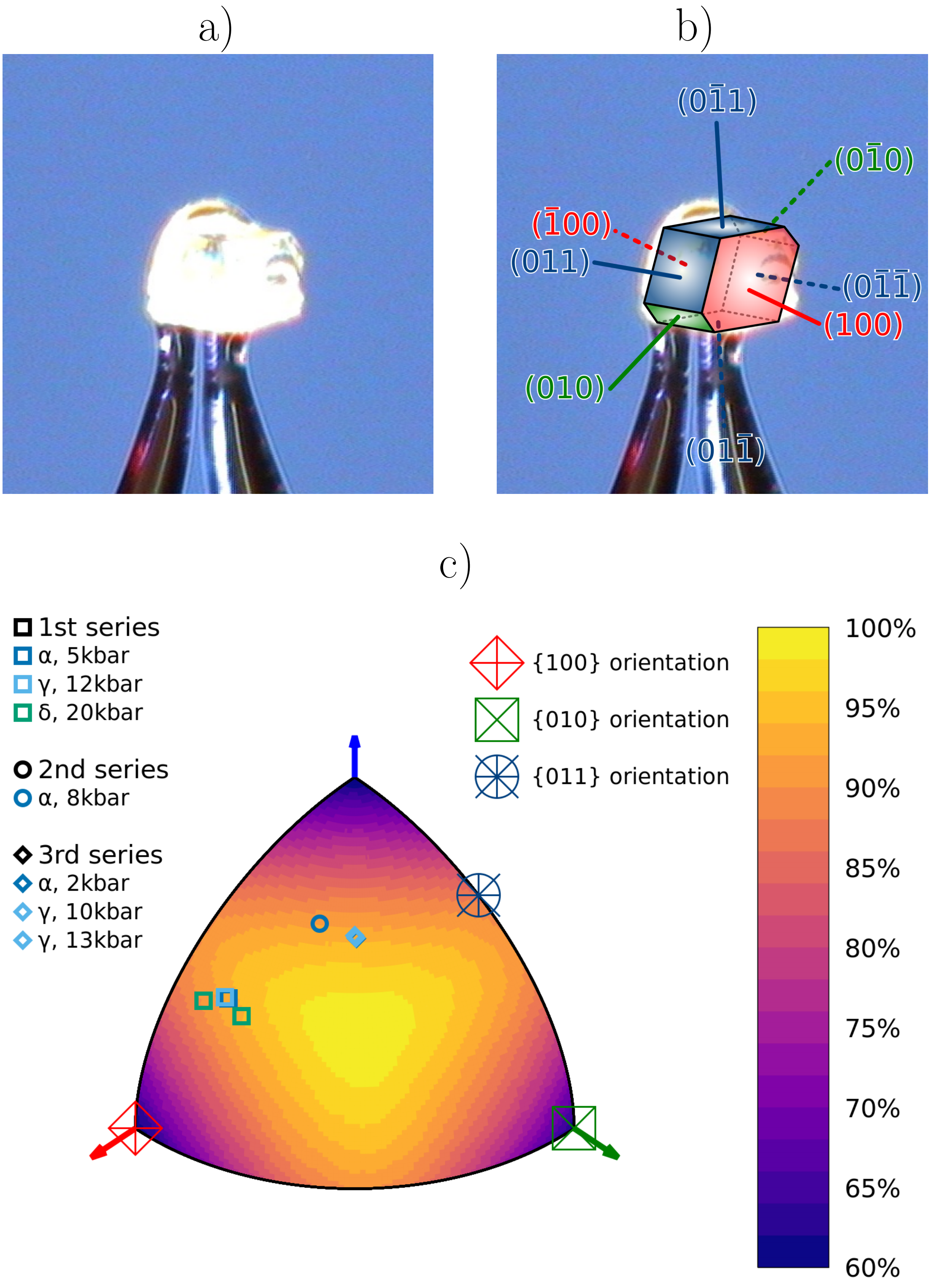


***Figure S1.2:*** *Crystal specimen of 2°AP-α used in 3rd series of HP experiments, a) without and b) with its faces highlighted. c) A potency heatmap for 2°AP-α/γ from main text, with orientations corresponding to highlighted faces marked using large symbols. Orientations achieved in 3rd series of experiments, marked with rhombi ⋄, lie between* {100} *and* {011} *on the map. Such placement required balancing the crystal on the edge connecting faces* (100) *and* (011) *using epoxy glue.*

## S1.2 Space group choice

New-found high-pressure crystal phases 2°AP-$\gamma$ and 2°AP-$\delta$ were initially described in space groups #33 ($Pna2_1$) and #14 ($P2_1/c$) in their standard settings. For the sake of consistency this choice was changed for non-standard space group settings for new polymorphs. Individual 2°AP molecules were placed in the same region of unit cell in all 8 solved structures. A comparison of unit cells in standard and selected space group settings was presented in Table S1.5.

***Table S1.5:*** *Standard and selected unit cell setting for 2°AP-$\alpha$, $\gamma$ and $\delta$.*

| | Standard setting | Selected setting |
|---|---|---|
| 2°AP-$\alpha$ | SG #62 $P\frac{2_1}{n}\frac{2_1}{m}\frac{2_1}{a}$<br>unit cell 7 × 16 × 11Å<br>principal axis $c$ | SG #62 $P\frac{2_1}{n}\frac{2_1}{m}\frac{2_1}{a}$<br>unit cell 7 × 16 × 11Å<br>principal axis $c$ |
| 2°AP-$\gamma$ | SG #33 $Pna2_1$<br>unit cell 7 × 11 × 16Å<br>principal axis $c$ | SG #33 $Pn2_1a$<br>unit cell 7 × 16 × 11Å<br>principal axis $b$ |
| 2°AP-$\delta$ | SG #14 $P1\frac{2_1}{c}1$<br>unit cell 16 × 11 × 7Å<br>principal axis $b$ | SG #14 $P11\frac{2_1}{a}$<br>unit cell 7 × 16 × 11Å<br>principal axis $c$ |

One difficult task at hand was to identify the polymorph $\gamma$ and subsequently choose the appropriate space group while solving collected datasets. As mentioned in the main text, space groups $Pnma$ and $Pn2_1a$ feature exactly the same extinctions rules, while strong similarity between both crystal structures makes any distinction based on intensity statistics surprisingly hard. Nonetheless it can be proved that an additional polymorph between orthorhombic 2°AP-$\alpha$ and monoclinic 2°AP-$\delta$ exists and crystal structures collected in the pressure range between approximately 9 and 18 kbar cannot be well described using only $Pnma$ or $P112_1/a$ symmetry.

An overview of selected data reduction and refinement statistics have been presented in Table S1.6. When investigating the phase transition one's first instinct would suggest looking at sharp changes in unit cell parameters. While they are very clear in transition to monoclinic crystal system of $\delta$, the trend for $a$, $b$, $c$ and $V$ as a function of pressure shows no clear shift or even kink visible (see S4.2, full dots).

Normalized intensity statistic $< |E^2 - 1| >$ should assume value of 0.968 in idealized centrosymmetric and 0.736 in idealized non–crentrosymmetric space group. Since $Pnma$ is centrosymmetric while $Pn2_1a$ is not, we could expect high value of the statistic for 2°AP-$\alpha$ and low for 2°AP-$\gamma$. In seven datasets featuring orthorhombic cell from Table S1.6 the value of $< |E^2 - 1| >$ is consistently close to its upper theoretical limit without regard for presence or absence of inversion center. This most probably stems from the fact, that the tilt of 2°AP molecule leading to the loss of $m_y$ mirror plane can occur in either direction, resulting in a sample which is twinned, with twin law mimicking the former $m_y$ mirror plane, as well as from the coexistence of the traces of $\delta$ alongside $\gamma$ phase, contaminating the intensities of

low-resolution reflections. We can see a slight decrease between 0.78 and 0.97 kbar, however such a small difference could not be a deciding factor while choosing correct symmetry.

Merging statistic $R_{int}$ is not more useful. Its value is consistently lower for $Pn2_1a$ compared to $Pnma$, as the extinction rules for both groups are the same, while in the latter more reflections are being averaged due to higher point group symmetry ($m2m$ versus $mmm$).

Only in direct space we can see discrepancy between crystal structure of 2°AP-$\alpha$ and 2°AP-$\gamma$. In first four datasets the R-factors remain similar between data solved in $Pn2_1a$ and $Pnma$. They are expectedly better for lower symmetry setting, but the difference is not very large. That is, unless we look at datasets 5–7, where a sharp increase of R-factors in $Pnma$ is observed. While crystal structure of 2°AP-$\alpha$ can be well-described in the space group of 2°AP-$\gamma$ (as $Pn2_1a$ is sub-group of $Pnma$), the reverse is not true and results in $R1 > 15\%$ and $wR2 > 35\%$ in those cases.

The differences in GooF between $Pnma$ and $Pn2_1a$ are, again, not large. However, their values tend to be closer to unity whenever a correct space group is chosen. Datasets 1–4 feature too many independent parameters when solved in $Pn2_1a$, while datasets 5–7 have too little free parameters when solved in $Pnma$.

Finally, for crystal structures solved in space group $Pn2_1a$ we can monitor the numerical value of 2°AP molecule rotation around axis z, "side-tilt", which constitutes the biggest difference between $\alpha$ and $\gamma$, as discussed in section 3.3 of main text. This value is fixed by symmetry to 0 in space group $Pnma$, relatively close to 0 in four datasets incorrectly solved in $Pn2_1a$, and much further from 0 in three datasets which should not be described in $Pnma$. This tilt appears suddenly somewhere around 9kbar, and shows a clear need for distinguishing separate polymorph $\gamma$, acting as a missing link between known 2°AP-$\alpha$ and skew monoclinic 2°AP-$\delta$.

***Table S1.6:*** *Selected data reduction and independent atom model refinement statistics for seven datasets which feature orthorhombic unit cell.*

| Polymorph | $\alpha$ | $\alpha$ | $\alpha$ | $\alpha$ | $\gamma$ | $\gamma$ | $\gamma$ |
|---|---|---|---|---|---|---|---|
| Pressure \ GPa | 0.00 | 0.25 | 0.52 | 0.78 | 0.97 | 1.16 | 1.30 |
| $a$ \ Å | 7.21024 | 7.2462 | 7.150 | 7.0642 | 7.0300 | 7.0202 | 6.9714 |
| $b$ \ Å | 16.4876 | 16.536 | 16.17 | 16.220 | 16.147 | 16.0775 | 16.139 |
| $c$ \ Å | 11.2792 | 11.255 | 11.288 | 11.166 | 11.1214 | 11.1610 | 11.072 |
| $<\|E^2-1\|>$ | 1.052 | 1.054 | 1.075 | 1.039 | 0.945 | 1.010 | 0.941 |
| in $Pnma$: | | | | | | | |
| Rint | 0.0949 | 0.1567 | 0.0719 | 0.1095 | 0.1048 | 0.1323 | 0.1514 |
| R1 | 0.0921 | 0.1376 | 0.1057 | 0.1410 | 0.2172 | 0.2204 | 0.2480 |
| R1 ($I > 2\sigma$) | 0.0547 | 0.0627 | 0.0499 | 0.0640 | 0.1632 | 0.1572 | 0.1820 |
| wR2 | 0.1506 | 0.1700 | 0.1292 | 0.1818 | 0.3876 | 0.3707 | 0.5439 |
| wR2 ($I > 2\sigma$) | 0.1385 | 0.1393 | 0.1100 | 0.1495 | 0.3662 | 0.3456 | 0.5260 |
| GooF | 1.029 | 0.997 | 0.980 | 1.025 | 1.088 | 1.080 | 1.845 |
| $Pna2_1$: | | | | | | | |
| Rint | 0.0915 | 0.1517 | 0.0691 | 0.1058 | 0.1033 | 0.1248 | 0.1402 |
| R1 | 0.0903 | 0.1360 | 0.1067 | 0.1629 | 0.1323 | 0.1502 | 0.1895 |
| R1 ($I > 2\sigma$) | 0.0513 | 0.0592 | 0.0488 | 0.0770 | 0.0652 | 0.0753 | 0.0895 |
| wR2 | 0.1380 | 0.1612 | 0.1340 | 0.2807 | 0.1616 | 0.1997 | 0.2627 |
| wR2 ($I > 2\sigma$) | 0.1246 | 0.1295 | 0.1119 | 0.1974 | 0.1388 | 0.1626 | 0.2190 |
| GooF | 0.983 | 0.986 | 0.947 | 0.971 | 1.022 | 0.992 | 1.007 |
| 2°AP side-tilt | 0.322(17)° | 0.41(7)° | 0.36(7)° | 0.58(12)° | 4.38(4)° | 5.03(7)° | 4.31(7)° |

## S1.3 Deposition

Structures mentioned in this paper were deposited as individual entries within Cambridge Structural Database. [Groom et al., 2016] The deposition numbers assigned to all discussed final models: 4 structures of 2°AP-$\alpha$ (one in low temperature and three under high pressure), 3 structures of 2°AP-$\gamma$ and a structure of 2°AP-$\delta$ solved using twinned diffraction data, were assigned deposition numbers CCDC 2096512–2096519. Exact numbers for each structure have been presented in Table S1.7.

***Table S1.7:*** *CCDC deposition numbers of crystal structures investigated in these studies, sorted by crystal phase and exerted pressure.*

| Polymorph | Pressure | Temperature | CCDC deposition number |
|---|---|---|---|
| 2°AP-$\alpha$ | 0.00 GPa | 100K | 2096512 |
| | 0.25 GPa | room temperature | 2096513 |
| | 0.52 GPa | room temperature | 2096514 |
| | 0.78 GPa | room temperature | 2096515 |
| 2°AP-$\gamma$ | 0.97 GPa | room temperature | 2096516 |
| | 1.16 GPa | room temperature | 2096517 |
| | 1.30 GPa | room temperature | 2096518 |
| 2°AP-$\delta$ | 2.03 GPa | room temperature | 2096519 |

# Chapter S2

# Structure description

## S2.1 Phase transitions

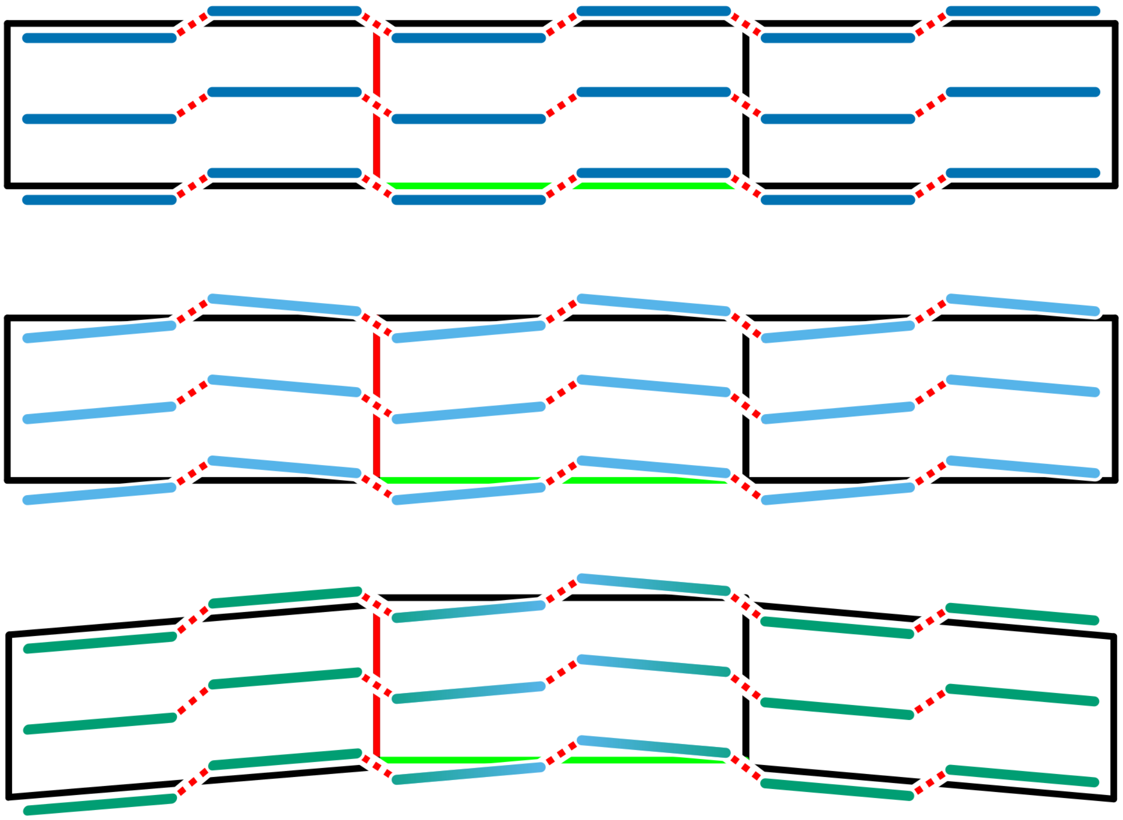

***Figure S2.1:*** *Schematic representation of three consecutive unit cells of 2°AP-α (top), 2°AP-γ (middle), and twinned 2°AP-δ (bottom) as seen from z. Fragments characteristic for each crystal phase have been colored dark blue, sky blue and green, respectively. Twinning during the γ → δ transition can be explained via collapse of two domains of orthorhombic 2°AP-γ (represented here by left- and right-most unit cell) in different directions.*

# Chapter S3

# Lattice interactions

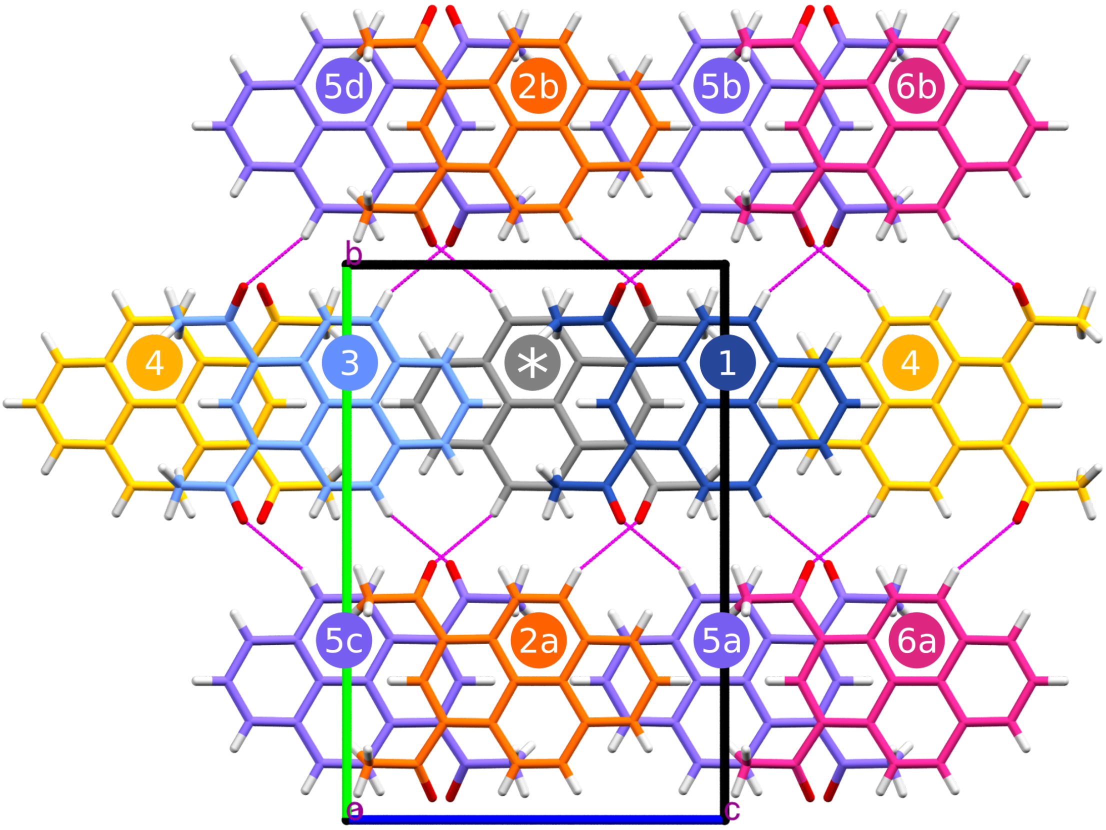


***Figure S3.1:*** *Cluster of molecules observed in 2°AP-$\alpha$, $\gamma$ and $\delta$ along x. In agreement with methodology utilized during Crystal Explorer calculations [M. J. Turner et al., 2017], only molecules with contacts shorter then 3.8Å to central molecule (colored gray, marked with $*$) were included. Moieties were colored and numbered according to interaction shared with central molecule in 2°AP-$\alpha$. Label suffixes were introduced whenever same-interactions in $\alpha$ split to sub-types in lower symmetric $\gamma$ or $\delta$. Further description and energy of interactions was presented in Tables S3.1 and S3.2.*

The energy of interactions was estimated using CrystalExplorer version 17.5. [M. J. Turner et al., 2017, Turner et al., 2014]

***Table S3.1:*** *Additional description of interactions present in 2°AP-α, γ and δ, visualized in Figure S3.1 and further evaluated in Table S3.2.*

| | dominating interaction | interacting or closest atoms | symmetry relation in α | γ | δ |
|---|---|---|---|---|---|
| Interaction 1 | $\pi\cdots\pi$, C–H$\cdots\pi$ | C18–H18A$\cdots\pi$<br>C24–H24A$\cdots\pi$ | $a_z$, $2_{1x}$ | $a_z$ | $a_z$ |
| Interaction 2 | short C–H$\cdots$O | C10–H10$\cdots$O1<br>O4$\cdots$H4–C4 | $\bar{1}$, $2_{1y}$ | $2_{1y}$ | $\bar{1}$ |
| Interaction 3 | $\pi\cdots\pi$ | C7$\cdots$C16 | $a_z$, $2_{1x}$ | $a_z$ | $a_z$ |
| Interaction 4 | dispersive | C6–H6$\cdots$C18<br>C8–H8$\cdots$C24 | $t_z$ | $t_z$ | $t_z$ |
| Interaction 5 | dispersive | C18–H18C$\cdots$C10<br>C24–H24B$\cdots$C4 | $n_x$, $2_{1z}$ | $n_x$ | $2_{1z}$ |
| Interaction 6 | long C–H$\cdots$O | C18–H18C$\cdots$O4<br>C24–H24B$\cdots$O1 | $\bar{1}$, $2_{1y}$ | $2_{1y}$ | $\bar{1}$ |

***Table S3.2:*** *Energy of interactions found in 2°AP at investigated pressure points. Listed values were calculated in Crystal Explorer and expressed in kJ mol$^{-1}$. Equivalence of selected interactions was marked using either vertical alignment or by linking equivalent interactions with a vertical curve. In agreement with methodology utilized during Crystal Explorer calculations, only interactions with paths shorter then 3.8Å were included. Visualization and further description of interactions was presented in Figure S3.1 and Table S3.1.*

| Crystal phase<br>Pressure \ GPa | α<br>0.00 | α<br>0.25 | α<br>0.52 | α<br>0.78 | γ<br>0.97 | γ<br>1.16 | γ<br>1.30 | δ<br>2.03 |
|---|---|---|---|---|---|---|---|---|
| Interaction 1 | -62.7 | -62.2 | -62.8 | -64.4 | -63.6 | -63.5 | -62.6 | -63.2 |
| Interaction 2a | -27.1 | -26.0 | -27.0 | -27.0 | -27.4 | -27.8 | -27.9 | -28.8 |
| Interaction 2b | | | | | | | | -27.3 |
| Interaction 3 | -21.4 | -21.2 | -22.0 | -22.3 | -22.5 | -22.5 | -22.7 | -23.6 |
| Interaction 4 | -10.2 | -10.2 | -10.4 | -11.0 | -11.5 | -11.2 | -11.7 | -11.6 |
| Interaction 5a | -8.1 | -8.2 | -9.0 | -9.5 | -9.8 | -9.5 | -10.2 | -10.1 |
| Interaction 5b | | | | | -9.9 | -10.0 | -10.1 | -11.2 |
| Interaction 5c | | | | | | | | -10.1 |
| Interaction 5d | | | | | -9.8 | -9.5 | -10.2 | -11.2 |
| Interaction 6a | -8.7 | -8.9 | -9.2 | -10.1 | -10.2 | -10.5 | -10.1 | -11.6 |
| Interaction 6b | | | | | | | | -8.8 |

# Chapter S4

# Theoretical calculations

A series of theoretical calculations in varying pressure conditions was performed for each reported crystal phase in CRYSTAL17 [Dovesi et al., 2018]. Dispersion corrected DFT calculations utilized B3LYP hybrid functional and 6-31G** basis set, the combination of which has already proved to give reliable results for pyrene derivatives during earlier work. Strict convergence criteria for Coulomb and Exchange sums were applied $(10^{-7}, 10^{-7}, 10^{-7}, 10^{-7}, 10^{-29})$.

At first an attempt to perform a series of calculations using EOS routine already implemented in CRYSTAL was made. [Erba et al., 2014] This approach firstly optimizes input structure at 0 kbar, after which it limits the volume of resulting crystal (by 15% here, as defined by user) and then performs multiple optimization steps in ever-increasing volume. In case of 2°AP this approached failed, as the structure of 2°AP-$\delta$ in high-pressure conditions would not converge when using low-pressure coordinates as a starting point – the molecules would instead tilt in the other direction, leading to a non-physical geometry and lack of convergence.

In order to avoid comparing results obtained in different way, three series of calculations (one for each polymorph) in decreasing volume conditions were prepared manually instead. Firstly, the volume of each unit cell at 0 kbar was estimated. Then an optimization for 85% of $V_0$ was performed, but instead of importing atomic coordinates from the ambient-pressure calculations, they were taken directly from XRD model instead. Using this approach, crystal structures of all three polymorphs at high pressure could be obtained without any issues. Afterwards, the volume was gradually increased (in $2\%V_0$ steps), and an optimization was performed at each volume step up to $105\%V_0$. Values of unit cell volume and total crystal energy at resulting 11 points were fit to a Vinet equation of state [Vinet et al., 1987] using the Levenberg-Marquardt algorithm in custom Python script. In case of $\alpha$ and $\gamma$, obtained results were in good agreement with the outcome of the automated procedure. Visualization and detailed results of the fit were presented in Figure S4.1 and Table S4.1.

Out of three structures discussed in this text, only specimen of 2°AP-$\alpha$ were obtained from multitude of performed crystallization attempts. This can be seen as somewhat puzzling in the context of calculations, as they suggest the $\alpha$ phase to feature the lowest stability out of three measured polymorphs, even in low pressure domain. 2°AP-$\delta$ and 2°AP-$\gamma$ were estimated to be $1 - 2\ \text{kJ mol}^{-1}$ more stable at

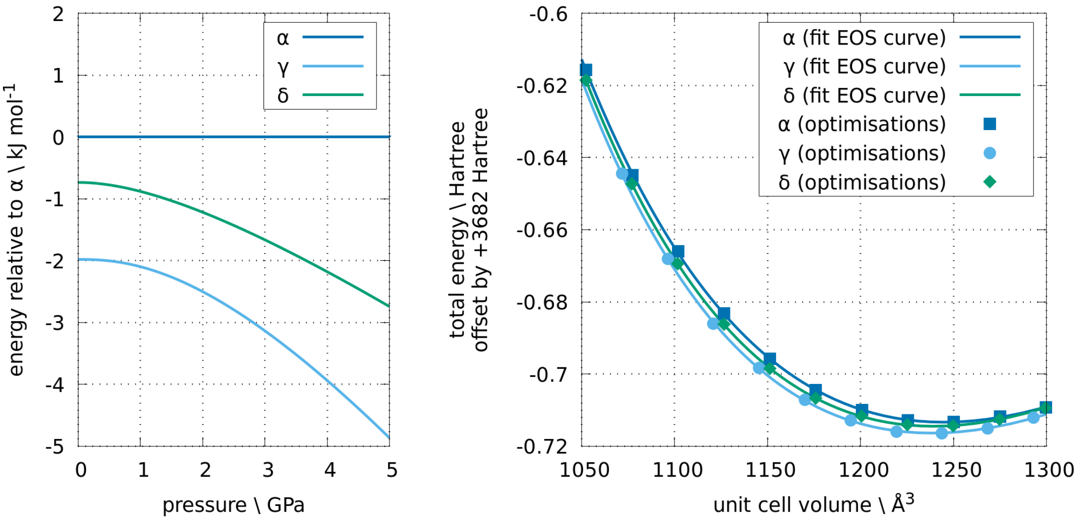


***Figure S4.1:*** *Summary of fitting optimized crystal structures to Vinet equation of state [Vinet et al., 1987]. Left: fit energy of polymorphs relative to 2°AP-α expressed as a function of pressure. Right: total energy of unit cell as a function of its volume.*

0 GPa and 2 – 4 kJ mol$^{-1}$ more stable at 4 GPa. It should be, however, noted that the calculations were performed at 0 Kelvin and assuming no thermal motions. Although including thermal motion is expected to notably influence the final results, it was deemed excessive for the scope of this work. The discord between calculations and experiment might also suggest a low kinetic stability of $\gamma$ and $\delta$, which would somewhat explain the instability of $\delta$'s 85%$V_0$ optimization as discussed above.

***Table S4.1:*** *Detailed results of fitting theoretically optimized bulk structures of 2°AP-α, γ and δ to Vinet equation of state. [Vinet et al., 1987]*

| Structure | 2°AP-$\alpha$ | 2°AP-$\gamma$ | 2°AP-$\delta$ |
|---|---|---|---|
| $V_0$ \ Å$^3$ | 1244.7682460755 | 1238.2703872981 | 1239.2221600955 |
| $E_0$ \ AU | -3682.7133282350 | -3682.7163436187 | -3682.7144480839 |
| $B_0$ \ GPa | 17.2483403125 | 17.2906185135 | 17.7739121193 |
| $B_0'$ \ 1 | 8.0922209551 | 8.9723515596 | 8.4508494643 |

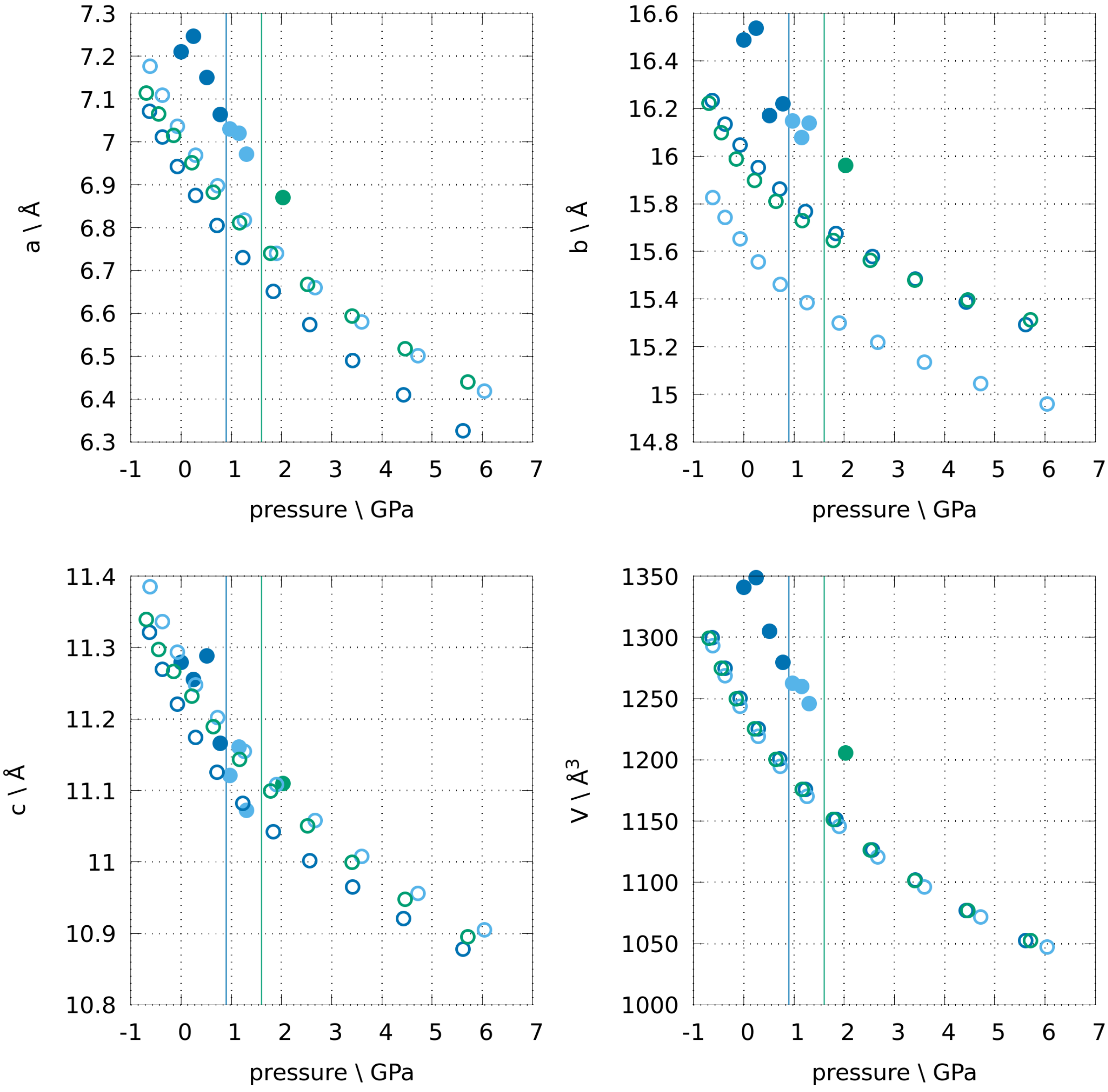


***Figure S4.2:*** *Evolution of 2°AP experimental (full circles) and optimized (empty circles) unit cell parameters with pressure. Polymorphs $\alpha$, $\gamma$ and $\delta$ are represented using dark blue, sky blue and green markers, respectively. Predicted values of pressure at which phase transitions occurs were marked using vertical lines.*

# Chapter S5

# Completeness

## S5.1 Generating potency maps

An overview of methodology used by *Hikari* to calculate potency and generate its maps have been presented in Section 2.3 of the main text. Unless the calculations are performed for a specific crystal, the program assumes a large unit cell with parameters $a = b = c = 20$Å, $\alpha = \beta = 90°$ and $\gamma = 90°$ (120° in hexagonal lattice). For crystals belonging to trigonal system, a hexagonal setting is used.

Choosing large unit cell in direct space populates the reciprocal space with more lattice points. This effectively increases numerical precision, and subsequently limits effects associated with multiple reflections entering / leaving the available region at the same time, which otherwise would be visible as "ripples" on the map. Since such behaviour makes the maps more smooth, it is desirable for the purpose of discerning general trends, rather than exactly calculating obtainable completeness for specifically defined conditions (as described below).

Prediction of potency for a single orientation such as the one performed for 2°AP in Table 2 of main text are the simplest type of estimation performed by *Hikari*, as they require only one – known – orientation and are performed for a constant unit cell, wavelength and resolution. In this approach a ratio between number of experimental and theoretically-possible reflections can be compared to obtain not only potency, but also applicable completeness. Other types of calculations require more though put into their design.

An example of non-trivial *Hikari* routine is presented on Figure 3 of the main text. There, the software was used to estimate the reciprocal space coverage in various experimental conditions. As such, its function was limited to a (very slow) numerical integration. The Figure and associated data have been obtained using the following steps:

1. Prepare a list of $\Theta$ angles of interest (close to 0°, equal 10°, 20°...);
2. Make an evenly spaced list of DAC opening angles (0° to 90° every 1°);
3. For each $\Theta$ and opening angle, estimate average potency in point group 1;
4. For each $\Theta$ visualize the potency as a function of opening angle.

Two further applications of *Hikari* requiring their own sophisticated routine are violin distribution plots and potency maps, examples of which are presented below on Figures S5.1 – S5.6. The violin plots are prepared based on distribution of potency calculated for a series of 50'000 orientation points each. Crystal orientations are selected in the following way: firstly four special orientations [100], [010], [001] and [111] are considered, as they correspond to maximal and minimal potency in multiple laue classes; then the 49'996 remaining points are selected quasi-uniformly from the unit sphere using an appropriate Fibonacci lattice. [González, 2010]

The approach warrants that each region of the sphere is equally represented. However, it is important to note that crystals are rarely spherical, and the Miller indices of their faces usually are small integers. Therefore, while predicting optimal experimental set-up for an experiment, one should not rely on the violin plots, but rather dedicated potency maps.

The potency maps require a different set of orientations than violin plots to be calculated. Both tools used to visualize the data (`matplotlib` and `gnuplot`) require the heatmaps to be probed uniformly in the selected reference frame. The "heat" of each point represents potency at a given orientation. Since here the heatmap is prepared in spherical coordinates, sample orientations have to be probed every $\theta = 1°$ and $\varphi = 1°$ around selected axis z. As this latitude–longitude lattice offers coverage which is very anisotropic and over-represented on the poles, it is not used to calculate overall completeness statistics presented on violin plots, as discussed above.

A quick overview of available potency in the form of a "cheat sheet" for Mo $K\alpha$/ Ag $K\alpha$ and opening angles of $35°/55°$ have been presented on pages S22–S28. Each page contains visualisation of completeness as a function of crystal orientation for all laue classes, in a form of a violin plot and associated potency maps. Descriptive statistics of presented plots are present in Table S5.1.

Finally, it can be argued that potency maps generated for monoclinic crystal system are incomplete, as not octant, but rather a quarter of sphere is necessary in order to represent all unique orientations in reciprocal space. However, due to a high $\infty/mm$ symmetry of available region in reciprocal space, the potency in this crystal system in a function of angle between orientation-vector and y only. Hence, only an octant of sphere is presented for simplicity and consistency. Similarly, while some of the other maps contain redundant information, e.g. all orientations in Laue class $m\bar{3}m$ could be presented on 1/48th of the sphere instead of the octant, the investigated area have been kept constant for the sake of consistency.

***Table S5.1:*** *Descriptive statistics for potency distribution as a function of sample orientation for all 11 Laue classes. Calculated based on four datasets used to obtain violin distribution plots in the main text (Figure 4), as discussed above. Rows $q_1$ – $q_3$ contain quartiles, while row "std" standard deviation of the distribution.*

| | $\bar{1}$ | $2/m$ | $mmm$ | $4/m$ | $4/mmm$ | $\bar{3}$ | $\bar{3}m$ | $6/m$ | $6/mmm$ | $m\bar{3}$ | $m\bar{3}m$ |
|---|---|---|---|---|---|---|---|---|---|---|---|
| Mo $K\alpha$, $\sin\theta/\lambda = 0.6$Å$^{-1}$ ($\Theta \approx 25°$) 35° opening angle | | | | | | | | | | | |
| mean | 27.95 | 44.48 | 59.43 | 67.40 | 77.47 | 64.49 | 76.90 | 78.78 | 84.02 | 94.68 | 96.35 |
| std | 0.04 | 6.21 | 9.63 | 8.41 | 11.55 | 5.56 | 12.07 | 9.96 | 13.05 | 4.68 | 4.95 |
| min | 27.68 | 27.55 | 29.75 | 30.14 | 29.41 | 28.06 | 27.49 | 30.15 | 29.17 | 69.73 | 67.83 |
| $q_1$ | 27.92 | 41.30 | 52.19 | 62.18 | 71.81 | 65.62 | 69.67 | 74.86 | 79.09 | 92.37 | 95.24 |
| $q_2$ | 27.95 | 47.52 | 60.05 | 70.63 | 79.52 | 66.25 | 79.47 | 82.41 | 87.74 | 96.15 | 98.46 |
| $q_3$ | 27.97 | 49.22 | 67.87 | 74.37 | 86.28 | 66.75 | 86.61 | 86.10 | 93.49 | 98.26 | 99.53 |
| max | 28.21 | 50.02 | 74.05 | 75.64 | 93.19 | 69.51 | 95.38 | 87.49 | 98.58 | 99.74 | 100.00 |
| Mo $K\alpha$, $\sin\theta/\lambda = 0.6$Å$^{-1}$ ($\Theta \approx 25°$) 55° opening angle | | | | | | | | | | | |
| mean | 58.97 | 78.29 | 87.55 | 94.47 | 95.81 | 95.91 | 96.04 | 96.68 | 96.16 | 100.00 | 99.99 |
| std | 0.04 | 9.89 | 8.70 | 6.23 | 7.04 | 7.20 | 7.48 | 6.66 | 7.29 | 0.03 | 0.05 |
| min | 58.68 | 58.27 | 59.00 | 59.82 | 58.30 | 58.61 | 57.30 | 59.70 | 57.69 | 99.24 | 98.93 |
| $q_1$ | 58.94 | 69.99 | 82.27 | 92.99 | 95.20 | 96.19 | 95.89 | 96.94 | 95.95 | 100.00 | 100.00 |
| $q_2$ | 58.97 | 79.74 | 89.80 | 96.41 | 99.10 | 99.13 | 99.87 | 99.98 | 99.96 | 100.00 | 100.00 |
| $q_3$ | 59.00 | 87.78 | 94.35 | 98.62 | 99.95 | 99.60 | 100.00 | 100.00 | 100.00 | 100.00 | 100.00 |
| max | 59.12 | 90.75 | 99.47 | 99.62 | 100.00 | 100.00 | 100.00 | 100.00 | 100.00 | 100.00 | 100.00 |
| Ag $K\alpha$, $\sin\theta/\lambda = 0.6$Å$^{-1}$ ($\Theta \approx 20°$) 35° opening angle | | | | | | | | | | | |
| mean | 34.71 | 53.33 | 67.65 | 76.42 | 84.11 | 75.84 | 85.12 | 86.48 | 88.92 | 98.24 | 98.52 |
| std | 0.04 | 7.75 | 10.29 | 9.06 | 11.36 | 7.19 | 12.30 | 10.43 | 12.53 | 2.69 | 2.88 |
| min | 34.30 | 34.23 | 36.32 | 36.87 | 35.85 | 35.06 | 34.27 | 36.92 | 35.60 | 81.07 | 78.89 |
| $q_1$ | 34.69 | 47.90 | 60.76 | 70.51 | 78.88 | 77.10 | 79.32 | 83.80 | 84.87 | 97.66 | 98.53 |
| $q_2$ | 34.72 | 57.05 | 68.09 | 79.17 | 86.74 | 78.23 | 88.07 | 90.10 | 93.81 | 99.43 | 99.87 |
| $q_3$ | 34.74 | 59.70 | 76.04 | 84.22 | 92.56 | 78.91 | 94.70 | 93.77 | 97.69 | 99.92 | 100.00 |
| max | 35.16 | 60.94 | 84.66 | 86.09 | 98.52 | 82.43 | 100.00 | 95.39 | 100.00 | 100.00 | 100.00 |
| Ag $K\alpha$, $\sin\theta/\lambda = 0.6$Å$^{-1}$ ($\Theta \approx 20°$) 55° opening angle | | | | | | | | | | | |
| mean | 64.61 | 82.97 | 90.83 | 96.78 | 97.11 | 97.23 | 97.09 | 97.56 | 97.11 | 100.00 | 100.00 |
| std | 0.04 | 9.59 | 7.82 | 5.28 | 5.86 | 5.93 | 6.14 | 5.38 | 6.02 | 0.00 | 0.00 |
| min | 64.47 | 63.96 | 64.47 | 65.38 | 63.73 | 64.32 | 62.88 | 65.21 | 63.03 | 100.00 | 100.00 |
| $q_1$ | 64.59 | 74.97 | 86.13 | 96.78 | 97.58 | 98.08 | 97.77 | 98.40 | 97.61 | 100.00 | 100.00 |
| $q_2$ | 64.62 | 84.10 | 93.21 | 98.73 | 99.90 | 99.99 | 100.00 | 100.00 | 100.00 | 100.00 | 100.00 |
| $q_3$ | 64.64 | 91.87 | 97.08 | 99.75 | 100.00 | 100.00 | 100.00 | 100.00 | 100.00 | 100.00 | 100.00 |
| max | 64.76 | 95.87 | 100.00 | 100.00 | 100.00 | 100.00 | 100.00 | 100.00 | 100.00 | 100.00 | 100.00 |
| | $\bar{1}$ | $2/m$ | $mmm$ | $4/m$ | $4/mmm$ | $\bar{3}$ | $\bar{3}m$ | $6/m$ | $6/mmm$ | $m\bar{3}$ | $m\bar{3}m$ |

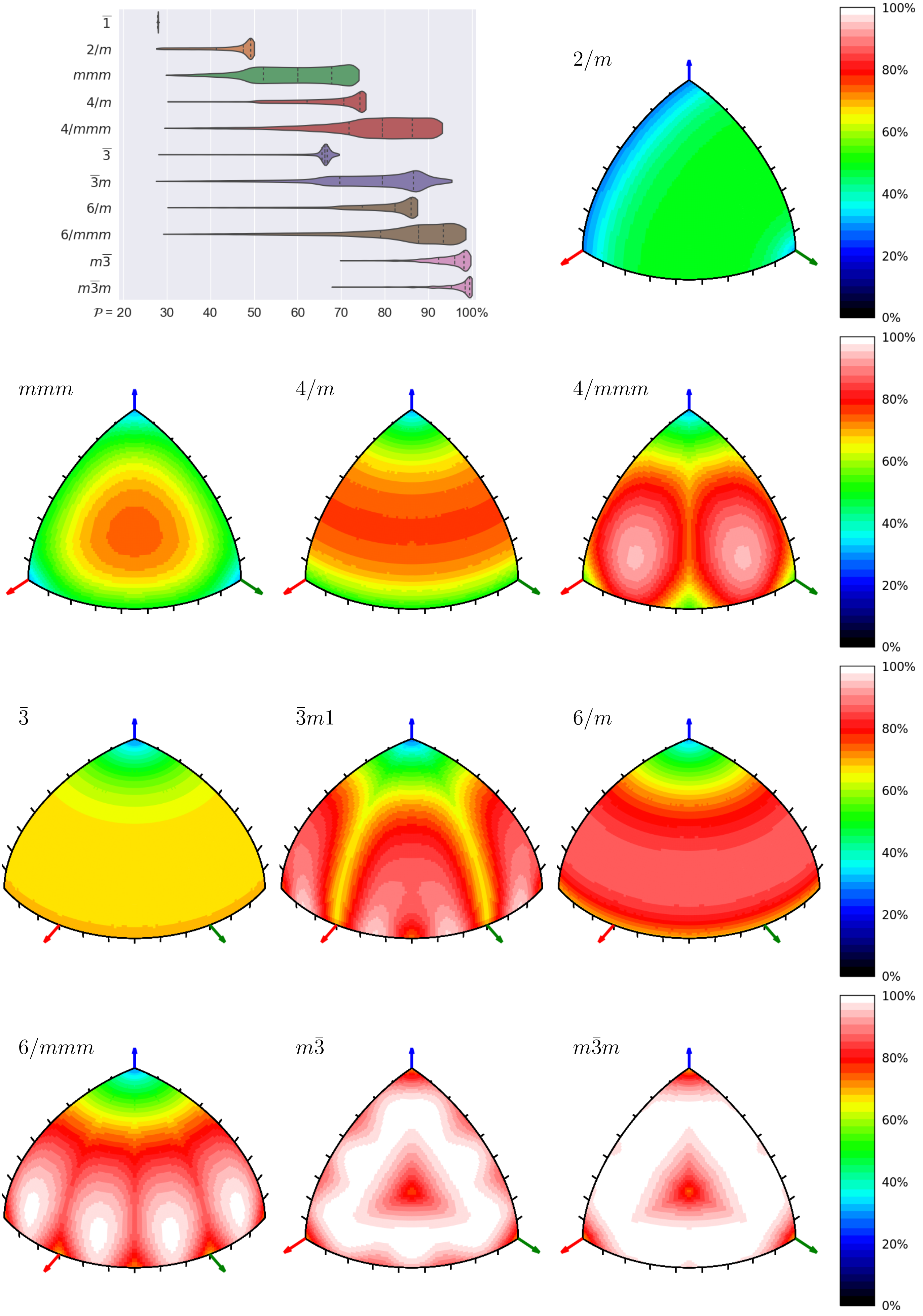


***Figure S5.1:*** *Potency maps to* $\sin\theta/\lambda = 0.6\text{Å}^{-1}$ *for Mo Kα and 35° opening angle. Tics every 10°.*

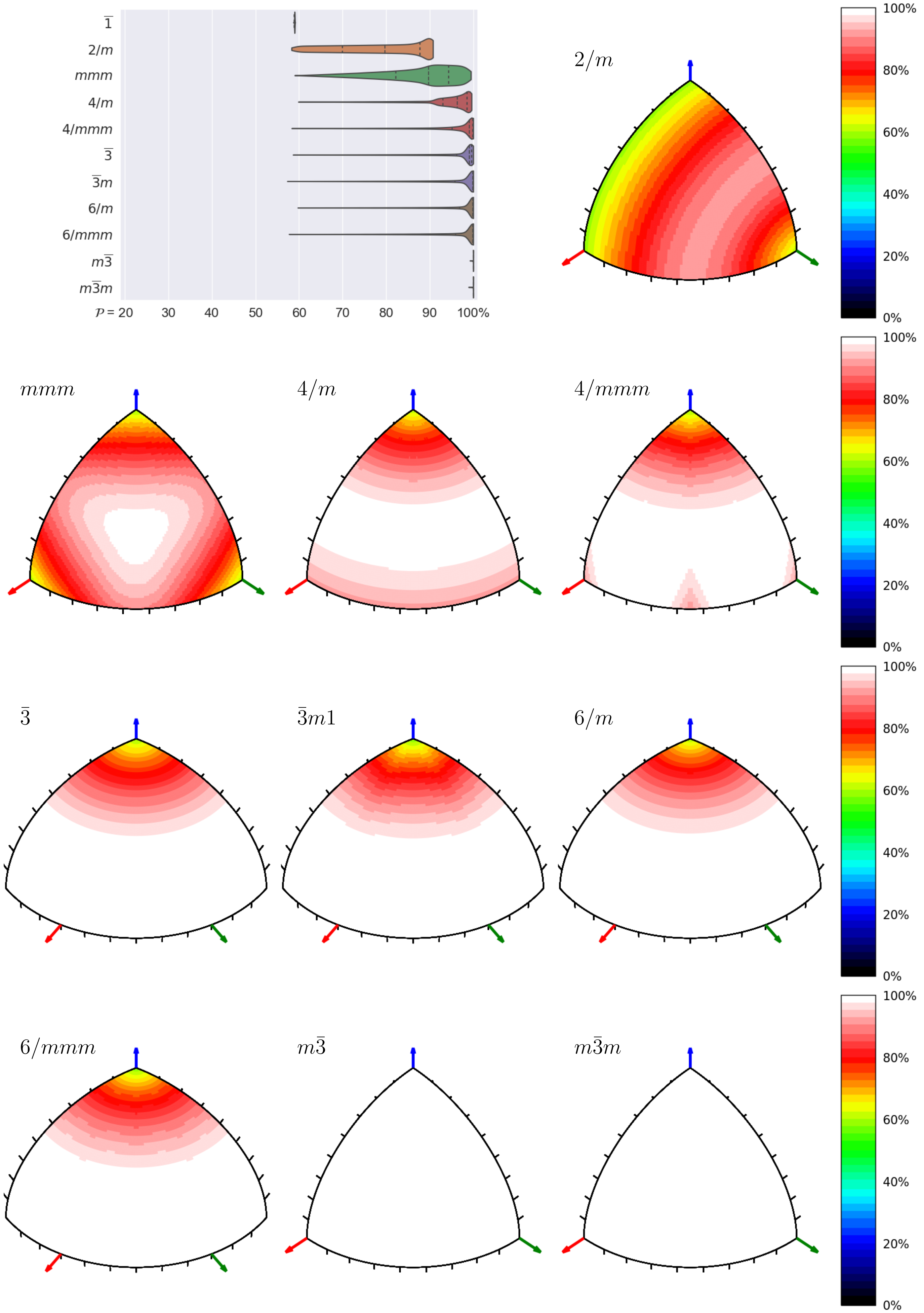


***Figure S5.2:*** *Potency maps to* $\sin\theta/\lambda = 0.6\text{Å}^{-1}$ *for Mo Kα and 55° opening angle. Tics every 10°.*

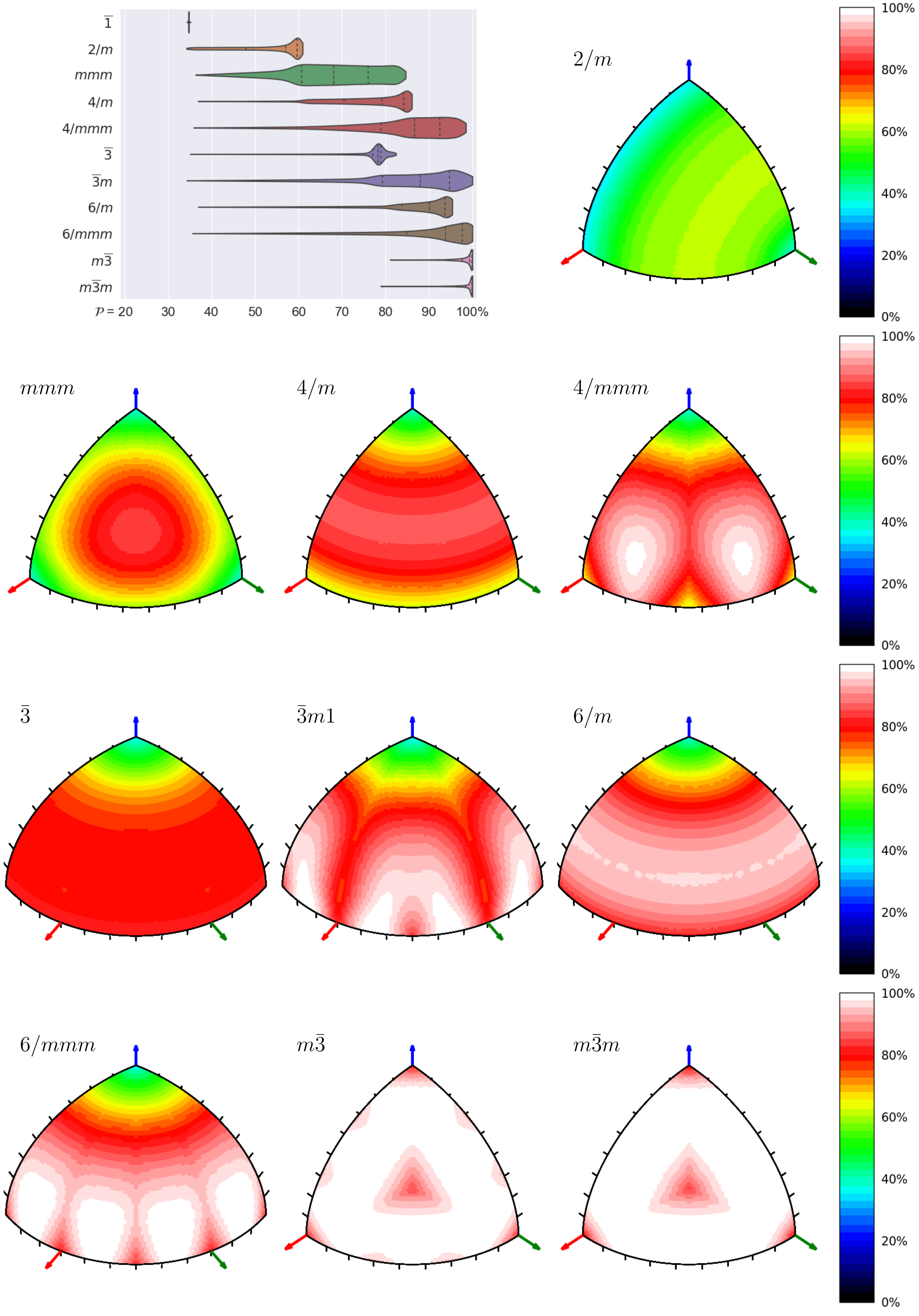


***Figure S5.3:*** *Potency maps to* $\sin\theta/\lambda = 0.6\text{Å}^{-1}$ *for Ag Kα and 35° opening angle. Tics every 10°.*

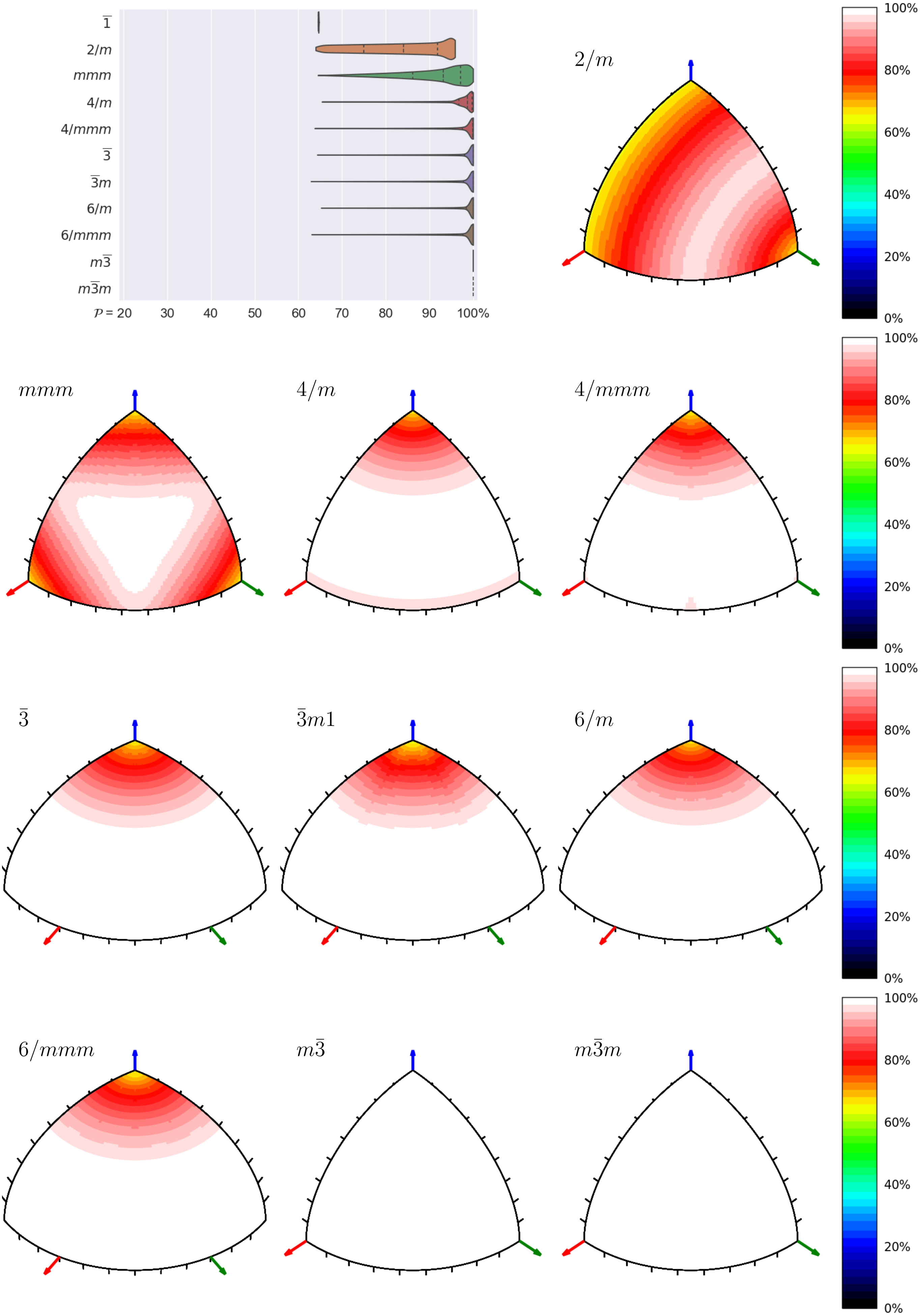


***Figure S5.4:*** *Potency maps to* $\sin\theta/\lambda = 0.6\text{Å}^{-1}$ *for Ag Kα and 55° opening angle. Tics every 10°.*

The following pages S27 and S28 contain potency maps which are quite different from other examples given in this work. It came to our attention that some researchers prefer not to interfere with crystal orientation and place samples on faces which warrant low potency, as such orientation allows a high coverage of specific reflection families: axes ($h00$, $0k0$, $00l$) and planes ($0kl$, $h0l$, $hk0$) which contain information about extinctions.

While high coverage of mentioned special elements is indeed important, it can be achieved even for slightly angled sample, especially if the aperture of Anvil Cell is large. Attentive sample placement in Laue classes $\bar{3}m$, $6/mmm$, $m\bar{3}$ and $m\bar{3}m$ can increase completeness of collected data from 30–70% to 100% while preserving full coverage of mentioned axes and planes even assuming Molybdenum $K\alpha$ radiation and 35° opening angle (abbreviated to "oa" below).

In order to show crystal orientations which preserve good coverage of special elements, special axes potency maps (page S27) and planes potency maps (page S28) have been prepared. These maps show the coverage of special regions using three color channels, one for each region: red for $h00/0kl$, green for $0k0/h0l$ and blue for $00l/hk0$. Total potency maps can be used in conjection with axes/planes potency maps to warrant both high overall completeness of data and good coverage of special positions.

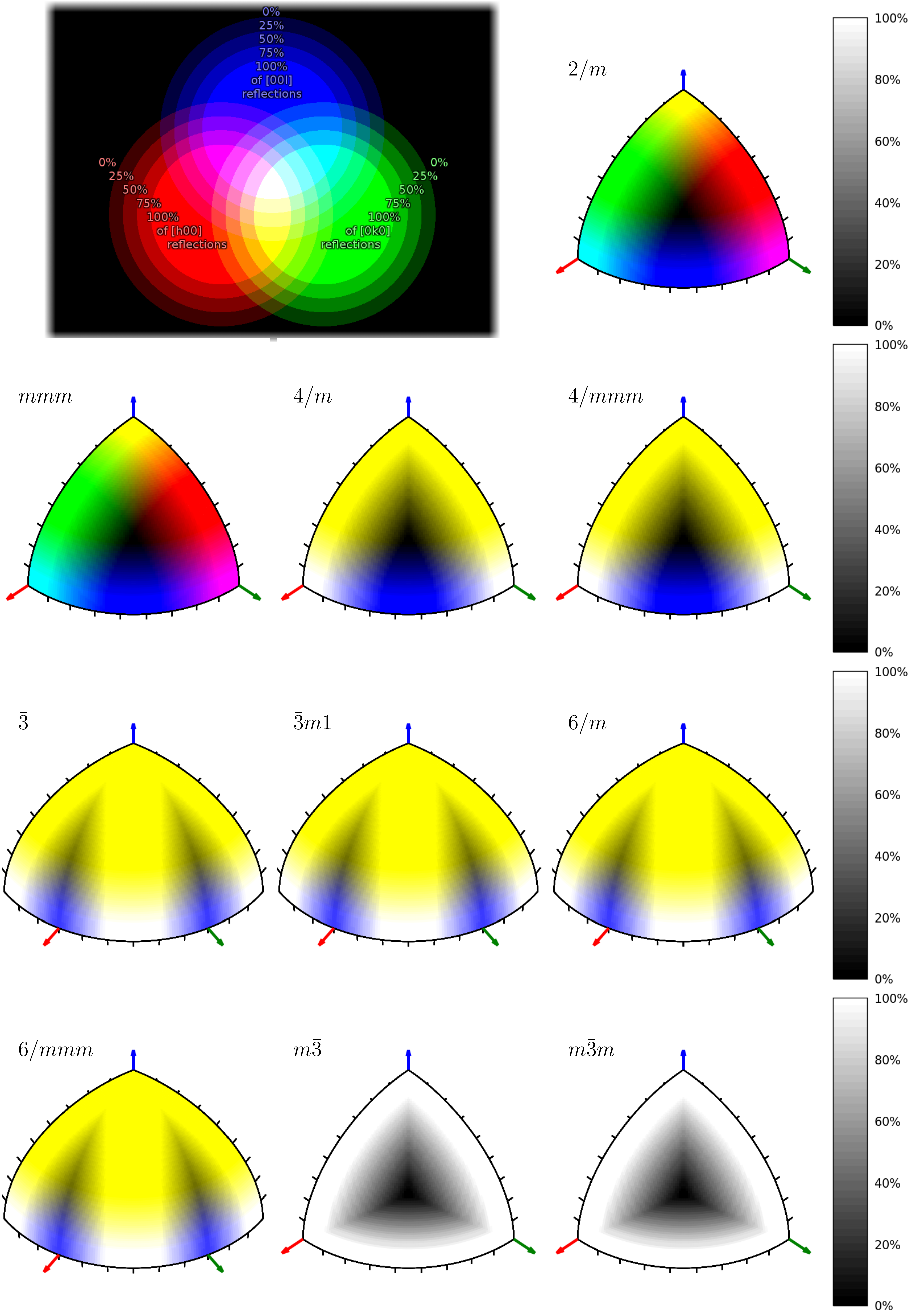


***Figure S5.5:*** *Axis potency maps to* $\sin\theta/\lambda = 0.6\text{Å}^{-1}$ *for Mo* $K\alpha$ *and 35° oa. Tics every 10°.*

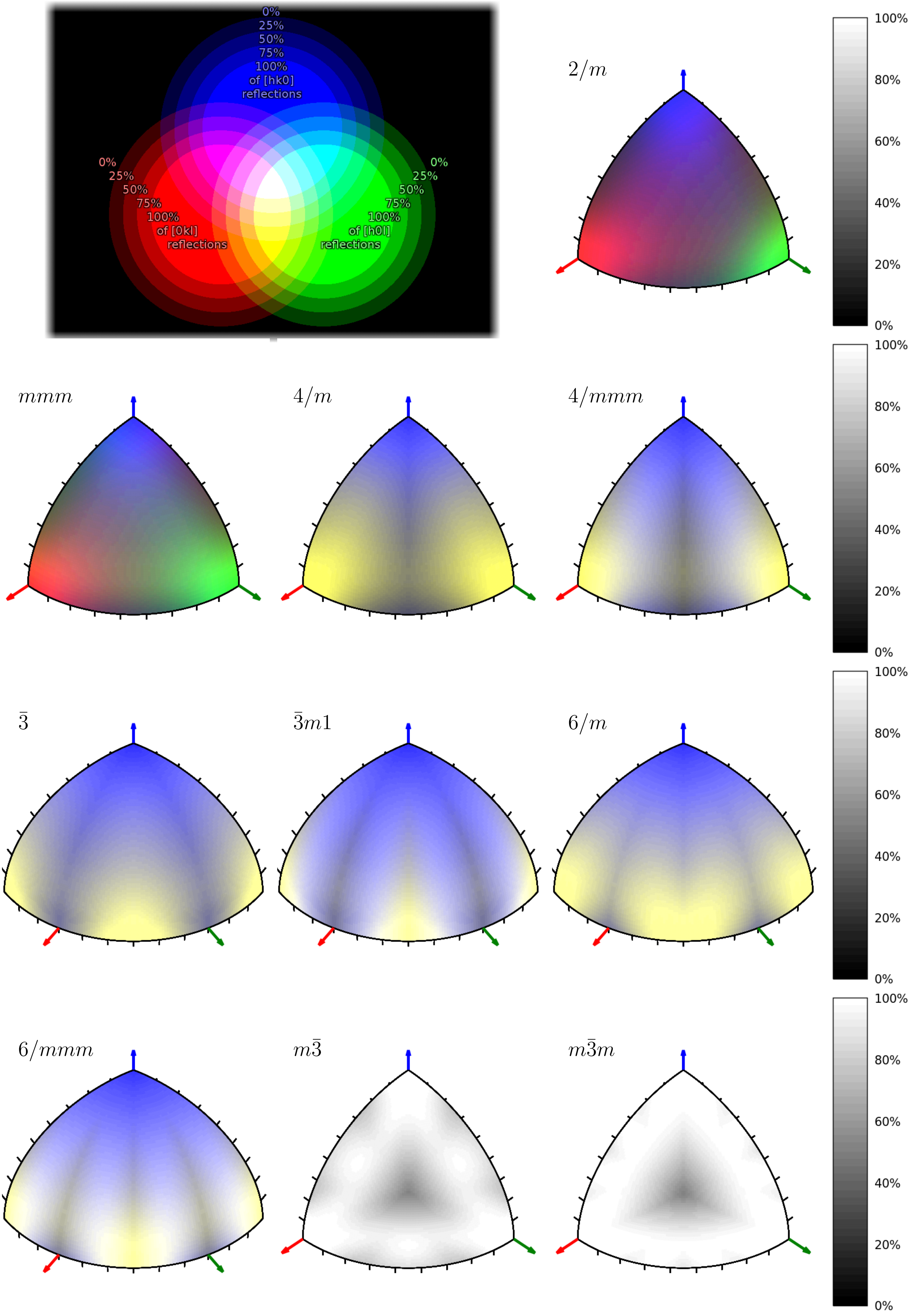


***Figure S5.6:*** *Plane potency maps to* $\sin\theta/\lambda = 0.6\text{Å}^{-1}$ *for Mo Kα and 35° oa. Tics every 10°.*

## S5.2 Reading potency maps

Potency maps can be essentially used in two ways: either to check what completeness is available using given sample orientation, or to find an orientation which warrants satisfactory completeness. Both applications are fairly simple and have been shortly introduced in the main text, yet they will be discussed below in more detail.

Let's assume a certain diffraction experiment has been performed - the crystal has been already placed within DAC and experimenter is interested in potency of this orientation or would like to slightly affect sample placement. In such a case, the face on which crystal has been placed has given Miller indices (hkl). If the face was natural the indices should be reducible to whole numbers, but if the sample was artificially tilted, crooked or cut, $h$, $k$ and $l$ might end up being any real numbers. In any case the Miller indices of diamond-perpendicular face can be calculated using UB orientation matrix:

$$(hkl)^T = (\mathbf{UB})^{-1} \begin{bmatrix} 1 \\ 0 \\ 0 \end{bmatrix} \tag{S5.1}$$

While Miller indices are a natural way to interpret crystal faces in crystallography and mineralogy, crystal face with given $(hkl)$ will be angled differently to internal symmetry elements in various crystals. Thus, the potency maps are prepared in cartesian coordinates. In order to obtain the Cartesian coordinates of a vector normal to the face $(hkl)$, the unit cell vectors must be considered:

$$\mathbf{x}^* = \left[\ \mathbf{a}^* \mid \mathbf{b}^* \mid \mathbf{c}^*\ \right] (hkl)^T = \mathbf{A}^*(hkl)^T \tag{S5.2}$$

After obtaining the vector $\mathbf{x}^*$ it is the most convenient to normalize it and use simple trigonometric relations to localize it on the potency map. For example, let's assume an orthorhombic crystal of 2°AP (cell parameters $a \approx 7$Å$, b \approx 16$Å$, c \approx 11$Å) has been placed in DAC on face with Miller indices $(01\bar{1})$. This fact could have been observed by trained eye of experimenter or calculated using **UB** matrix. Vector normal to $(01\bar{1})$ can be then obtained by:

$$\mathbf{x}^* = \left[\begin{array}{c|c|c} \frac{1}{7} & 0 & 0 \\ 0 & \frac{1}{16} & 0 \\ 0 & 0 & \frac{1}{11} \end{array}\right] \begin{bmatrix} 0 \\ 1 \\ -1 \end{bmatrix} = \begin{bmatrix} 0 \\ \frac{1}{16} \\ -\frac{1}{11} \end{bmatrix} \approx 0.11 * \begin{bmatrix} 0 \\ 0.57 \\ -0.82 \end{bmatrix} \tag{S5.3}$$

Now we can use vector $\mathbf{x}^*$ to find the completeness on relevant map for Laue class mmm. Firstly, since our map shows completeness only for positive values of $x$, $y$ and $z$, lets notice that faces $(01\bar{1})$ and $(011)$ (and thus directions $\left[\ 0 \ \ \frac{1}{16} \ \ -\frac{1}{11}\ \right]$ and $\left[\ 0 \ \ \frac{1}{16} \ \ \frac{1}{11}\ \right]$) are equivalent. Since in our case $x = 0$, $\mathbf{x}^*$ will point somewhere between axes $\mathbf{Y}^*$ and $\mathbf{Z}^*$, on the right edge of the map. As $0.57/0.82 \approx 0.7$, we can expect $\mathbf{x}^*$ to point slightly below the midpoint of this right edge; to be exact $\tan^{-1}(0.57/0.82) \approx 35°$ from axis $\mathbf{Y}^*$ towards axis $\mathbf{Z}^*$, signaling potency of $\approx 50\%$ for Mo $K\alpha$ radiation and DAC with lower aperture.

The opposite process, finding best orientation for given crystal is done using the same equations, but going the other way. The highest potency for Laue Class $mmm$ can be obtained for $\mathbf{x}^* = \begin{bmatrix} 1 & 1 & 1 \end{bmatrix}$. In order to translate it into $(hkl)$ indices, one has to multiply $\mathbf{x}^*$ by inverse of matrix $\mathbf{A}^*$ and reduce the result to whole numbers.

## S5.3 Opening angle

The definition of opening angle in community is unregulated and thus use of this term can be inconsistent. Some authors define opening angle $\alpha$ as angle between vector normal to diamond's surface and a limiting vector which glides along metal body of DAC (purple line on Figure S5.7a). Then its value tends to be close to 35° and can theoretically vary between 0 and 90°. Others use the term to describe maximum value of $2\theta$ obtainable in diffraction experiment, in which case typical and maximum $\alpha$ equal 70 and 180°, respectively. In this paper we use the term "opening angle" or "single opening angle" to express the first value, while terms "double opening angle" or occasional "aperture" are designated to express the second.

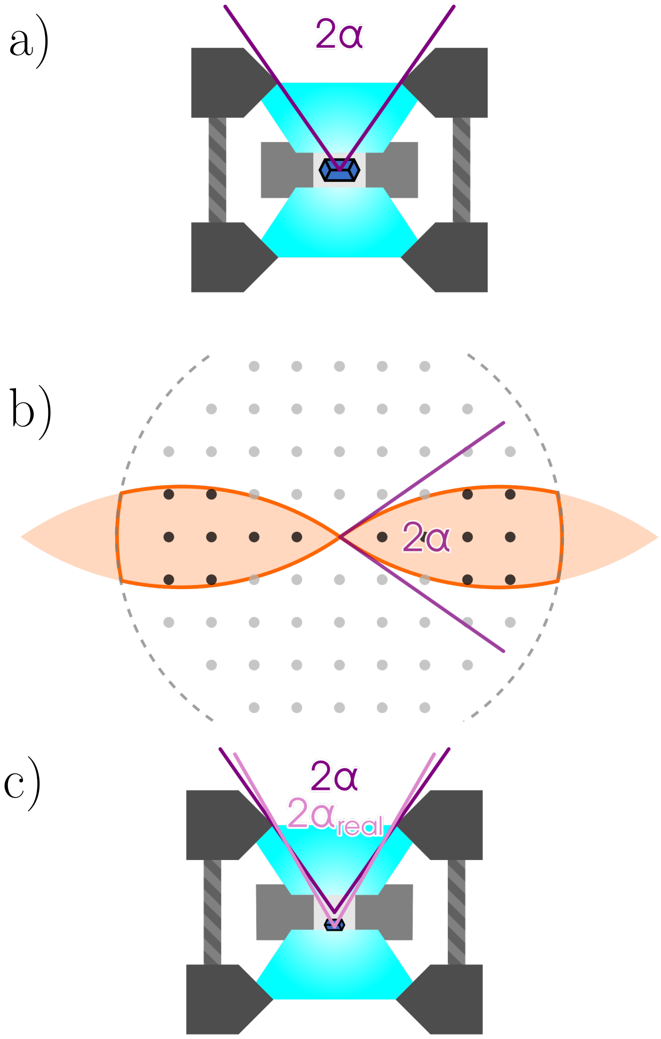


***Figure S5.7:*** *Double opening angle $2\alpha$ as it appears in a) direct and b) reciprocal space. Definition of $\alpha$ used in this paper is more practical during the description of reciprocal spacefor example if $\alpha > \Theta$, then the available disc is trimmed due to experimental resolution constraint (orange outline on b)). c) It is worth noting that "real" experimental opening angle can deviate from the nominal DAC value if the gasket is too thick or lies too close to the sample.*

# Chapter S6

# Bibliography

[Dovesi et al., 2018] Dovesi, R., Erba, A., Orlando, R., Zicovich-Wilson, C. M., Civalleri, B., Maschio, L., Rérat, M., Casassa, S., Baima, J., Salustro, S., and Kirtman, B. (2018). Quantum-mechanical condensed matter simulations with CRYSTAL. *Wiley Interdisciplinary Reviews: Computational Molecular Science*, 8(4):e1360.

[Erba et al., 2014] Erba, A., Mahmoud, A., Belmonte, D., and Dovesi, R. (2014). High pressure elastic properties of minerals from ab initio simulations: The case of pyrope, grossular and andradite silicate garnets. *J. Chem. Phys.*, 140(12):124703.

[González, 2010] González, A. (2010). Measurement of areas on a sphere using Fibonacci and latitude-longitude lattices. *Mathematical Geosciences*, 42(1):49–64. arXiv: 0912.4540.

[Groom et al., 2016] Groom, C. R., Bruno, I. J., Lightfoot, M. P., and Ward, S. C. (2016). The Cambridge Structural Database. *Acta Crystallographica Section B*, 72(2):171–179.

[M. J. Turner et al., 2017] M. J. Turner, J. J. McKinnon, S. K. Wolff, D. J. Grimwood, P. R. Spackman, D. Jayatilaka, and M. A. Spackman (2017). CrystalExplorer17 (2017).

[Turner et al., 2014] Turner, M. J., Grabowsky, S., Jayatilaka, D., and Spackman, M. A. (2014). Accurate and Efficient Model Energies for Exploring Intermolecular Interactions in Molecular Crystals. *The Journal of Physical Chemistry Letters*, 5(24):4249–4255.

[Vinet et al., 1987] Vinet, P., Smith, J. R., Ferrante, J., and Rose, J. H. (1987). Temperature effects on the universal equation of state of solids. *Phys. Rev. B*, 35(4):1945–1953.
